# ANKRD1 aggravates renal ischaemia‒reperfusion injury via promoting TRIM25‐mediated ubiquitination of ACSL3

**DOI:** 10.1002/ctm2.70024

**Published:** 2024-09-17

**Authors:** Shangting Han, Jiayu Guo, Chenyang Kong, Jun Li, Fangyou Lin, Jiefu Zhu, Tianyu Wang, Qi Chen, Yiting Liu, Haochong Hu, Tao Qiu, Fan Cheng, Jiangqiao Zhou

**Affiliations:** ^1^ Department of Organ Transplantation Renmin Hospital of Wuhan University Wuhan China; ^2^ Department of Urology Renmin Hospital of Wuhan University Wuhan China; ^3^ Department of Nephrology Renmin Hospital of Wuhan University Wuhan China; ^4^ Key Laboratory of Medical Electrophysiology Ministry of Education and Medical Electrophysiological Key Laboratory of Sichuan Province, Collaborative Innovation Center for Prevention of Cardiovascular Diseases, Institute of Cardiovascular Research, Southwest Medical University Luzhou China

**Keywords:** ACSL3, ANKRD1, ferroptosis, renal ischaemic‒reperfusion injury, TRIM25, ubiquitination

## Abstract

**Background:**

Renal ischaemia‒reperfusion injury (IRI) is the primary cause of acute kidney injury (AKI). To date, effective therapies for delaying renal IRI and postponing patient survival remain absent. Ankyrin repeat domain 1 (ANKRD1) has been implicated in some pathophysiologic processes, but its role in renal IRI has not been explored.

**Methods:**

The mouse model of IRI‐AKI and in vitro model were utilised to investigate the role of ANKRD1. Immunoprecipitation‐mass spectrometry was performed to identify potential ANKRD1‐interacting proteins. Protein‒protein interactions and protein ubiquitination were examined using immunoprecipitation and proximity ligation assay and immunoblotting, respectively. Cell viability, damage and lipid peroxidation were evaluated using biochemical and cellular techniques.

**Results:**

First, we unveiled that ANKRD1 were significantly elevated in renal IRI models. Global knockdown of ANKRD1 in all cell types of mouse kidney by recombinant adeno‐associated virus (rAAV9)‐mitigated ischaemia/reperfusion‐induced renal damage and failure. Silencing *ANKRD1* enhanced cell viability and alleviated cell damage in human renal proximal tubule cells exposed to hypoxia reoxygenation or hydrogen peroxide, while ANKRD1 overexpression had the opposite effect. Second, we discovered that ANKRD1's detrimental function during renal IRI involves promoting lipid peroxidation and ferroptosis by directly binding to and decreasing levels of acyl‐coenzyme A synthetase long‐chain family member 3 (ACSL3), a key protein in lipid metabolism. Furthermore, attenuating ACSL3 in vivo through pharmaceutical approach and in vitro via RNA interference mitigated the anti‐ferroptotic effect of *ANKRD1* knockdown. Finally, we showed ANKRD1 facilitated post‐translational degradation of ACSL3 by modulating E3 ligase tripartite motif containing 25 (TRIM25) to catalyse K63‐linked ubiquitination of ACSL3, thereby amplifying lipid peroxidation and ferroptosis, exacerbating renal injury.

**Conclusions:**

Our study revealed a previously unknown function of ANKRD1 in renal IRI. By driving ACSL3 ubiquitination and degradation, ANKRD1 aggravates ferroptosis and ultimately exacerbates IRI‐AKI, underlining ANKRD1's potential as a therapeutic target for kidney IRI.

**Key Points/Highlights:**

- Ankyrin repeat domain 1 (ANKRD1) is rapidly activated in renal ischaemia‒reperfusion injury (IRI) models in vivo and in vitro.
- ANKRD1 knockdown mitigates kidney damage and preserves renal function.
- Ferroptosis contributes to the deteriorating function of ANKRD1 in renal IRI.
- ANKRD1 promotes acyl‐coenzyme A synthetase long‐chain family member 3 (ACSL3) degradation via the ubiquitin‒proteasome pathway.
- The E3 ligase tripartite motif containing 25 (TRIM25) is responsible for ANKRD1‐mediated ubiquitination of ACSL3.

## Graphical abstract

In vivo and in vitro, ANKRD1 promptly responds to renal IRI.

ANKRD1 deficiency effectively alleviates renal injury and protects renal function.

Ferroptosis plays a key role in the function of ANKRD1 during renal IRI.

ANKRD1 facilitates the degradation of ACSL3 through the ubiquitin‒proteasome pathway.

TRIM25 mediates the K63‐linked ubiquitination of ACSL3, a process initiated by ANKRD1.

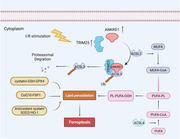

## 1 BACKGROUND

Renal ischaemia‒reperfusion injury (IRI) is the primary cause of acute kidney injury (AKI) and commonly occurs during kidney transplantation, partial nephrectomy and other clinical situations.^1^ Insufficient recuperation from AKI is regarded as contributing to the progression of chronic kidney disease and the ultimately progress to end‐stage renal failure. The main pathophysiological features and pathogenic events of renal IRI are renal tubular damage and death, involving mechanisms, such as free radical generation, inflammatory response, and ATP depletion.^2^, ^3^, ^4^ Currently, renal replacement therapy and nutritional supplementation are still the mainstays of AKI treatment in clinical practice.^5^ To date, the most effective therapy option for this renal disorder remains unavailable. Consequently, it is imperative to uncover novel therapeutic targets and provide safer and more effective therapeutic agents for AKI.

ANKRD1, which is highly conserved across mammals, is found on chromosome 10 of humans.^6^, ^7^ Previous research on ANKRD1 has mainly focused on its functions in cardiology and cancer. ANKRD1 is recognised to perform critical functions in the physiological and pathological ventricular remodelling, mechanosensing, regulation of gene expression and intracellular signalling.^7^, ^8^ Recent studies uncovered that ANKRD1 as a stress response protein, also functions in trauma and tissue damage.^9^, ^10^ It is noteworthy that *ANKRD1* was identified as the most significantly differentially expressed gene (DEG) in calcium oxalate crystal‐treated human proximal renal tubular epithelial (HK‐2) cells, suggesting a likely involvement of ANKRD1 in the regulation of renal injurious diseases. Nevertheless, the precise function of ANKRD1 in AKI is elusive.

Ferroptosis, resulting from the accumulation of lethal toxic membrane lipid peroxides induced by the labile iron pool overloading, has been identified as a major pathomechanism of renal IRI.^11^ Peroxidation of specific lipids exceeds the defense of the antagonistic system.^12^ As crucial enzymes, the long‐chain fatty acyl coenzyme A (CoA) synthetase (ACSLs) family plays a pivotal role in lipid metabolism. By accelerating the oxidation of fatty acids, ACSLs produces acyl‐CoA, with ACSL4 and ACSL3 being implicated in ferroptosis.^13^ ACSL4 is essential for the process of ferroptosis, as it causes increased lipid peroxidation and subsequent ferroptosis.^14^ Conversely, ACSL3 is a key molecule to monounsaturated fatty acids (MUFA)‐mediated ferroptosis resistance. ACSL3 substantially enhances MUFA activation, which limits the generation of lipid reactive oxygen species (ROS) by replacing polyunsaturated fatty acids (PUFA) to change cell membrane properties.^13^ Tripartite motif containing 25 (TRIM25), an E3 ligase, is responsible for catalyzing the ubiquitination and degradation of target proteins.^15^ Further examination is required to determine whether TRIM25 is also involved in renal IRI.

This present study provided the first evidence that ANKRD1 promotes renal tubular injury during IRI‐AKI via its direct interaction with TRIM25 to modulate TRIM25‐mediated ubiquitination of ACSL3 in renal proximal tubule cells (RPTCs), leading to excessive lipid peroxidation and triggering the activation of ferroptosis. Our research provided insight into the underlying mechanisms of IRI‐AKI and highlighted the possible targets for mitigating kidney IRI.

## 2 MATERIALS AND METHODS

### 2.1 Animals and treatment

Male C57BL/6 mice (20−22 g, 8 weeks old) were obtained from the Center of Experimental Animals at Wuhan University Medicine College. They were provided unrestricted access to a standard laboratory feed and water and were kept at 20°C−22°C with a 12‐h light‒dark cycle.

The renal IRI model was established as described previously.^16^ Please refer to the Supporting Information and Methods for details.

### 2.2 Gene delivery in vivo

Recombinant adeno‐associated virus 9 (rAAV9) was produced by Genechem was utilised to modulate the expression of ANKRD1 or TRIM25 in mouse kidney. A shRNA oligo was subcloned into a GV478 AAV serotype 9 vector (U6‐MCS‐CAG‐EGFP) to develop the rAAV9‐shANKRD1. Target sequence of ANKRD1 in mice: GTTCAGAAATGGGAGAATATGA. Wild‐type male mice were injected with rAAV9 into kidneys 3 weeks before ischaemia/reperfusion (I/R) surgery. An amount of 40 µL rAAV9‐shANKRD1 or control rAAV9‐vector (total 4 × 10^11^ v.g./mouse) was injected into bilateral kidneys of anaesthetised mice using a microlitre syringe. A needle was inserted from the lower pole to the upper pole of the kidney, and rAAV9 solution was injected while the needle was slowly drawn out.^17^, ^18^, ^19^, ^20^, ^21^, ^22^, ^23^ enhanced green fluorescent protein (EGFP) detection was utilised to reflect the distribution of rAAV9 in the kidney. The method described above can also be applied to knockdown TRIM25. The target sequence for TRIM25 in mice is CGTGAAAGTCATCTTCGACTA.

### 2.3 Cell culture

HK‐2 cells (Cat. CL‐0109) and HEK 293T cells (Cat. CL‐0005) were purchased from Procell Life Science & Technology Co., Ltd. HK‐2 and HEK 293T cells were cultured in dulbecco's modified eagle medium (DMEM) (Gibco) supplemented with 10% fetal bovine serum (FBS) (Invitrogen) and 1% penicillin‒streptomycin, at 37°C in a 5% CO_2_ incubator. HK‐2 cells were treated with hydrogen peroxide (H_2_O_2_) or hypoxia reoxygenation (H/R) to establish two classical in vitro models of renal IRI according to previous studies.^24^, ^25^ HK‐2 cells were treated with vehicle or H_2_O_2_ (at a final concentration of 500 µM) for 6 h to construct in vitro models of renal IRI. For the H/R model, HK‐2 cells cultured in serum‐free medium were exposed to hypoxia for 48 h in a tri‐gas incubator (94% N_2_, 5% CO_2_ and 1% O_2_) and then reoxygenated for 6 h (95% air and 5% CO_2_).

### 2.4 Additional methods

Details of all procedures are presented in the Supporting Information.^26^

## 3 RESULTS

### 3.1 ANKRD1 expression is highly induced in renal IRI

**FIGURE 1:**
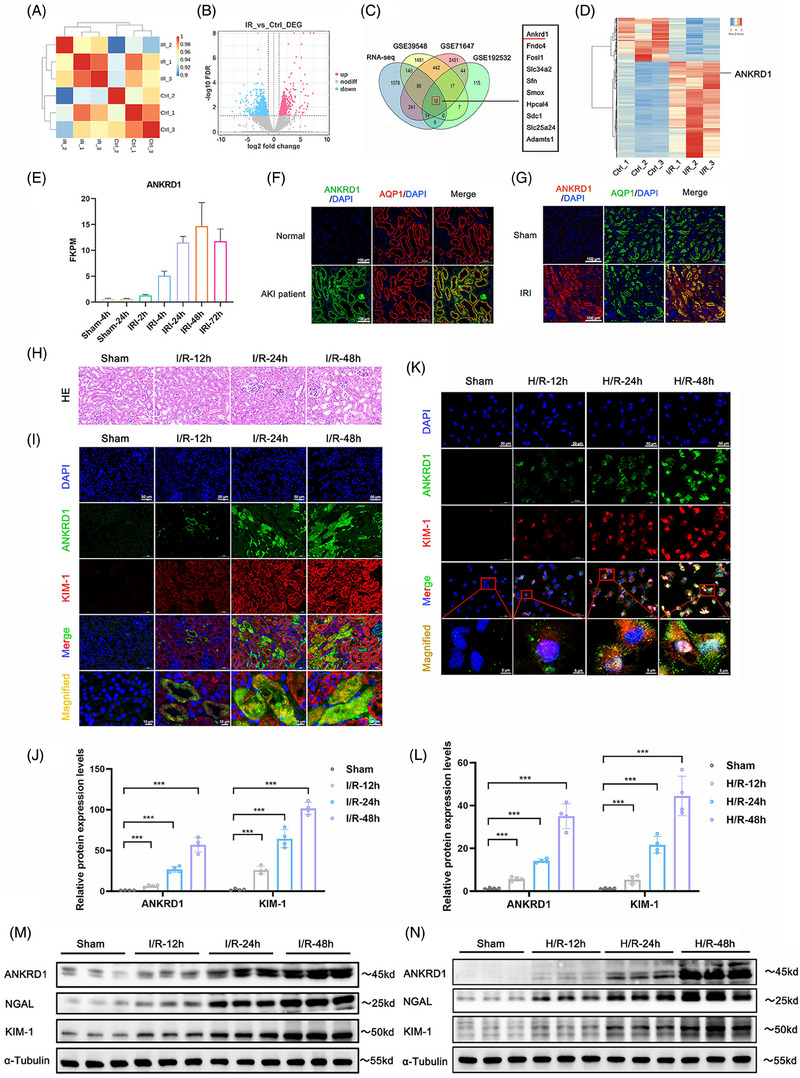
(A) Ankyrin repeat domain 1 (ANKRD1) expression is increased in renal ischaemia‒reperfusion injury (IRI) model in vivo and in vitro. Sample correlation analysis revealing that ischaemia/reperfusion (I/R)‐treated samples could be well distinguished from control. (B) The volcano plot showing 851 upregulated and 728 downregulated differentially expressed genes (DEGs). (C) The intersection of our RNA‐seq data with three renal IRI GEO datasets (https://www.ncbi.nlm.nih.gov/geo, GSE39548, GSE71647 and GSE192532), and 10 common genes including ANKRD1 were obtained. GSE39548 (*Mus musculus*): kidney_control group, *n* = 4; kidney_IRI group, *n* = 4. GSE71647 (*M. musculus*): wild‐type (WT) normal kidneys, *n* = 2; WT IRI kidneys, *n* = 2. GSE192532 (*M. musculus*): WT sham renal, *n* = 3; WT IRI 24 h renal, *n* = 3. (D) Heatmap showing DEGs after renal I/R treatment. (E) The transcriptome trend chart of ANKRD1 using data from GEO (https://www.ncbi.nlm.nih.gov/geo/query/acc.cgi, GSE98622). ANKRD1 expression peaked as blood flow reperfused the kidneys for 48 h. GSE98622 (*M. musculus*): sham‐4 h, *n* = 3; sham‐24 h, *n* = 3; IRI‐2 h, *n* = 3; IRI‐4 h, *n* = 3; IRI‐24 h, *n* = 3; IRI‐48 h, *n* = 3; IRI‐72 h, *n* = 3. (F and G) Co‐localisation of ANKRD1 and AQP1 in human renal biopsy specimens and kidney of mice. (H and I) Renal tissues were collected for haematoxylin and eosin (HE) staining and immunofluorescence (IF) staining of kidney injury molecule 1 (KIM‐1) and ANKRD1 expression. Scale bar, 50 µm. (J) Quantitative analysis of KIM‐1 and ANKRD1 positive staining. (K) The expression of ANKRD1 and KIM‐1 in H/R‐treated HK‐2 cells as determined by IF staining. Scale bar, 50 µm. (L) Quantitative analysis of ANKRD1 and KIM‐1‐positive staining. (M) Immunoblot analysis of ANKRD1, KIM‐1 and lipocalin 2 (NGAL) in kidney tissues with α‐tubulin as a loading control. (N) Immunoblot analysis of ANKRD1, KIM‐1 and NGAL in HK‐2 cells with β‐actin as a loading control. The data in (E‒G), (I), (J) and (L) are expressed as the mean ± standard deviation (SD). ^***^ *p* < .001.

To systematically identify master genes involved in renal IRI, we conducted transcriptome profiling to identify significant DEGs between I/R treated kidneys and sham kidneys in mice. Sample correlation analysis revealed a high degree of similarity within the experimental group, with clear differentiation between the I/R‐treated and control samples (Figure 1A). The volcano plot (Figure 1B) showed 851 upregulated and 728 downregulated DEGs. To narrow our research focus, we compared our datasets with three relevant renal IRI datasets (GSE39548, GSE71647 and GSE192532) from the GEO database, resulting in the identification of 10 overlapping DEGs (Figure 1C). Among these genes, *ANKRD1* exhibited sustained and significant upregulation in I/R‐treated kidneys (Figure 1D). The expression pattern of ANKRD1 during renal IRI was further validated using the published fourth relevant renal IRI dataset (GSE98622) from the GEO database. Notably, *ANKRD1* exhibited minimal expression in control kidneys, but its expression significantly increased (up to 18.4‐fold) following 48 h of reperfusion post‐ischaemia (Figure 1E). According to the kidney IRI scRNA‐seq data (https://susztaklab.com/Mouse_IRI_scRNA/Genemap.php) from Susztak and coworkers,^27^ the expression of ANKRD1 in mouse kidneys shows a significant increase in the proximal tubule epithelial cells (PT S3 and S1) after experiencing short (23 min) or long (30 min) bilateral ischaemia (Figure S3A,B). As shown by our co‐localisation of ANKRD1 with the marker protein AQP1, the ascending ANKRD1 is mainly localised in the renal tubular cells of human or mouse (Figure 1F,G).

To experimentally validate the results based on our transcriptome profiling and bioinformatics analysis, we established a mouse model of bilateral renal ischaemia followed by reperfusion for 12, 24 and 48 h. As the reperfusion time increased, serum blood urea nitrogen (BUN) and serum creatinine (SCr) levels significantly elevated (Figure S3C,D), accompanied by a marked increase in kidney injury as shown by our quantitative data (Figures 1H and S3E). Similar to the expression pattern of kidney injury molecule 1 (KIM‐1), a classical marker of early kidney injury, ANKRD1 expression increased over time in response to renal IRI and was localised to tubular epithelial cells and the renal interstitium (Figure 1I,J). On the other hand, we also established two classical in vitro models of renal IRI using HK‐2 cells exposed to H/R cycles and H_2_O_2_ challenge, respectively. The increasing of KIM‐1 confirmed the successful establishment of in vitro models, and the levels of ANKRD1 displayed a noticeable increase in the cytoplasm and nucleus of HK‐2 cells (Figure 1K,L). Consistent with two classical markers of early kidney injury, KIM‐1 and lipocalin 2 (NGAL), ANKRD1 levels in the in vivo/in vitro models of renal IRI consistently and significantly increased (Figures 1M,N and S3F).

### 3.2 Knockdown of ANKRD1 ameliorates AKI in vivo and in vitro

**FIGURE 2:**
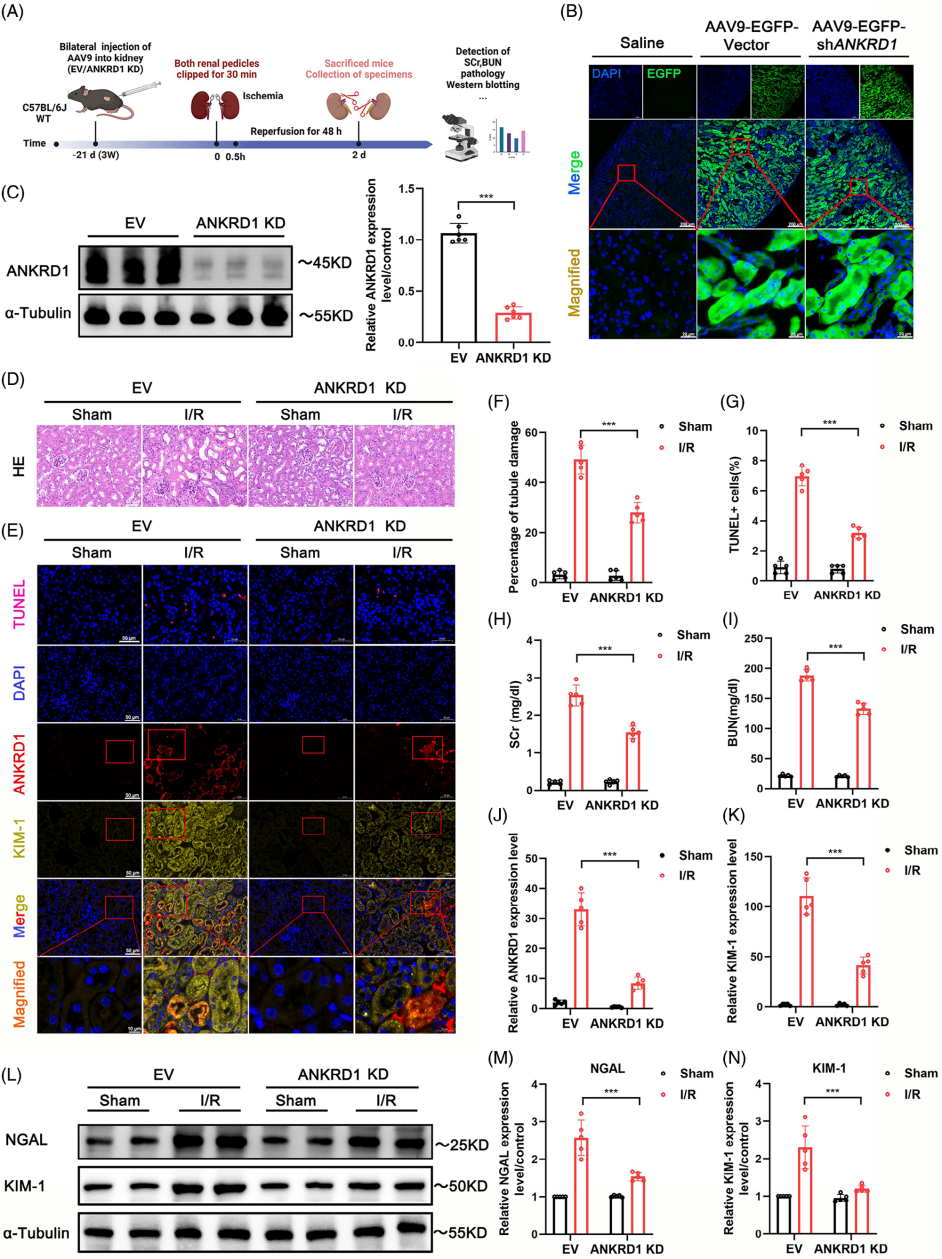
Conditional knockdown of ankyrin repeat domain 1 (ANKRD1) effectively alleviates renal injury and preserves function. (A) Schematic diagram of the animal experimental procedure. Created with Biorender.com. (B) The success of renal recombinant adeno‐associated virus (rAAV9) transduction was determined by EGFP fluorescence detection. Scale bar, 200 µm. (C) Immunoblotting of renal cortical tissues confirmed the knockdown efficiency of ANKRD1 protein. (D and E) Renal tissues were collected for haematoxylin and eosin (HE) staining, TUNEL assay and IF staining of kidney injury molecule 1 (KIM‐1) and ANKRD1 expression. Scale bar, 50 µm. (F) Quantitative analysis of tubular damage. (G) Quantitative analysis of TUNEL+ cells. (H and I) Serum creatinine (SCr) and blood urea nitrogen (BUN) measurement. (J and K) Quantitative analysis of ANKRD1 and KIM‐1‐positive staining. (L) Representative immunoblot images of KIM‐1 and lipocalin 2 (NGAL) in renal tissues. (M and N) Quantitative analysis of NGAL and KIM‐1. The data in (C), (E‒J), (L) and (M) are expressed as the mean ± standard deviation (SD). ^***^ *p* < .001.

To assess the therapeutic potential of ANKRD1 in the context of mouse IRI‐AKI, first, we utilised rAAV9‐mediated ANKRD1 knockdown strategy to inhibit ANKRD1 expression in mouse kidneys suffered from IRI. A schematic diagram illustrating the experimental procedure is presented in Figure 2A. The bilateral kidneys of mice were injected with either rAAV9 harbouring a targeting ANKRD1 shRNA expression cassette or a normal control rAAV9 using a microlitre syringe. After 3 weeks, both renal pedicles were clamped for 30 min and subsequently reperfused for 48 h. The appearance of bright green EGFP fluorescence in the kidney demonstrated the successful renal rAAV9 transduction (Figure 2B). The highly efficient knockdown of ANKRD1 in the renal cortical tissues was further validated through quantitative assessment of immunoblotting and immunofluorescence assays (Figure 2C,E,J). ANKRD1 knockdown protected against the renal tubular injury (Figure 2D,F), inhibited apoptosis of renal cortical tissues (Figure 2E,G), and decreased the abundances of KIM‐1 and NGAL in kidneys subjected to I/R treatment as evidenced by our immunoblotting and immunofluorescence data (Figure 2E,K‒N). Similarly, intravenous injection of shANKRD1 via the tail vein could alleviate renal IRI, manifested as the suppression of the increase in KIM‐1 levels (Figure S4). ANKRD1 knockdown mice exhibited significantly reduced levels of serum BUN and SCr, indicating remarkably improved renal function (Figure 2H,I).

**FIGURE 3:**
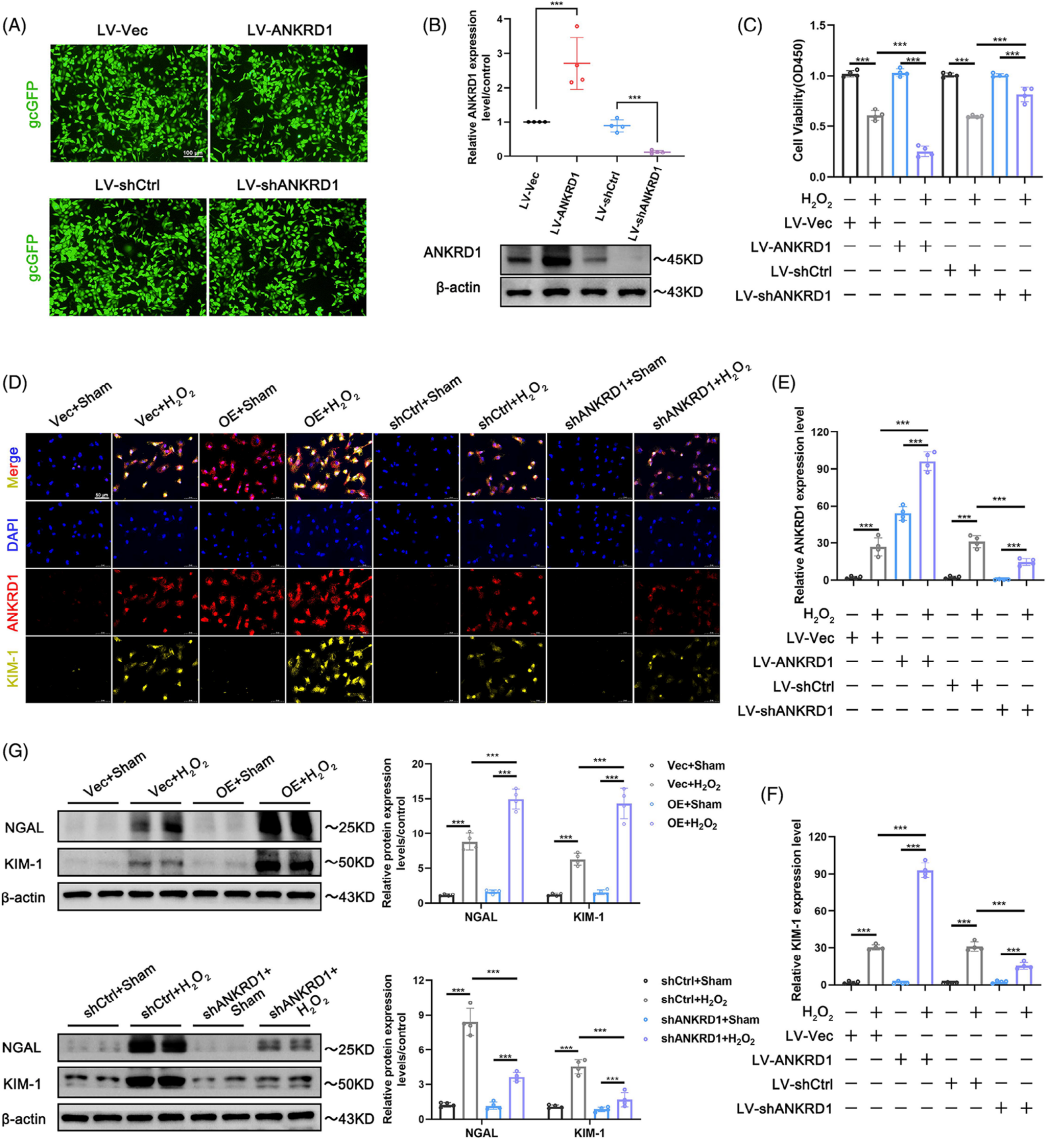
(A) Ankyrin repeat domain 1 (ANKRD1) inhibition rescues H_2_O_2_‐induced renal tubular epithelial cell damage and decreased viability. The success of lentivirus transduction was detected by gcGFP fluorescence. (B) Immunoblotting of HK‐2 cells confirmed the knockdown and overexpression efficiency of ANKRD1 protein. (C) Cell viability of HK‐2 cells under different treatment conditions was analysed by CCK‐8. (D) Representative cellular images of ANKRD1 and kidney injury molecule 1 (KIM‐1) IF staining. Scale bar, 50 µm. (E and F) Quantitative analysis of ANKRD1 and KIM‐1‐positive staining. (G) Representative immunoblot images and quantification of KIM‐1 and lipocalin 2 (NGAL) in HK‐2 cells. The data in (B), (C) and (E‒G) are expressed as the mean ± standard deviation (SD). ^***^ *p* < .001.

Subsequently, we employed a lentiviral vector tagged with gcGFP to knockdown or overexpress ANKRD1 in vitro (Figure 3A). The transfection effectiveness was validated through immunoblotting (Figure 3B). High levels of ANKRD1 decreased cell viability in response to H_2_O_2_ or H/R stimulation, whereas ANKRD1 knockdown improved the poor cell viability induced by H_2_O_2_ or H/R (Figures 3C and S5A). ANKRD1 overexpression further enhanced endogenous KIM‐1/NGAL abundance in HK‐2 cells subjected to H_2_O_2_ or H/R stimulation, whereas ANKRD1 knockdown effectively repressed the levels of KIM‐1/NGAL (Figures 3D‒G and S5B). Collectively, our immunoblotting and immunofluorescence data consistently demonstrated that ANKRD1 overexpression or knockdown can exacerbate or alleviate the accumulation of early kidney damage indicators, KIM‐1 and NGAL. These findings highlight ANKRD1 as a potential therapeutic target for IRI‐AKI.

### 3.3 Lipid peroxidation contributes to the deteriorating function of ANKRD1 during renal IRI and H_2_O_2_ and H/R treatment

**FIGURE 4:**
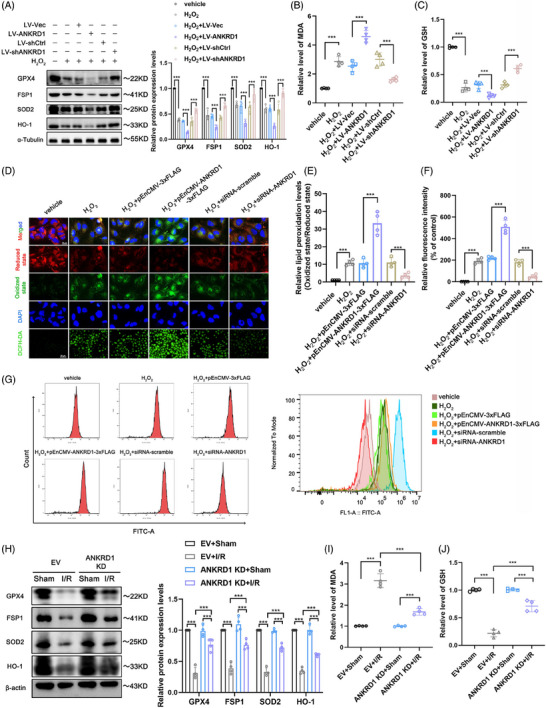
Lipid peroxidation contributes to the deteriorating function of ankyrin repeat domain 1 (ANKRD1) during renal ischaemia‒reperfusion injury (IRI) and H_2_O_2_ treatment. (A) Representative immunoblot images and quantification of GPX4, FSP1, HO‐1 and SOD2 in HK‐2 cells. (B and C) Intracellular malondialdehyde (MDA) and glutathione (GSH) levels of HK‐2 cells with different treatments. (D) Representative images of intracellular lipid peroxidation and total reactive oxygen species (ROS) in HK‐2 cells with different treatments. (E and F) Quantitative analysis of lipid peroxidation and total ROS. (G) Detection and quantification of ROS by flow cytometry. (H) Representative immunoblot images and quantification of GPX4, FSP1, HO‐1 and SOD2 in renal tissue. (I and J) MDA and GSH levels of renal tissue in each group. The data in (A‒C), (E), (F) and (H‒J) are expressed as the mean ± standard deviation (SD). ^***^ *p* < .001.

ANKRD1 was previously described as a possible regulator of ferroptosis^10^; nevertheless, its role in IRI organs has not been explored. In general, treatment with H_2_O_2_ and H/R resulted in a significant downregulation of anti‐ferroptosis proteins (GPX4, FSP1) and antioxidant proteins (SOD2 and HO‐1), as well as an accumulation of malondialdehyde (MDA) and a depletion of glutathione (GSH) in HK‐2 cells. ANKRD1 inhibition reduced these dysregulations, however high levels of ANKRD1 worsened the changes mentioned above (Figures 4A‒C and S6). In this study, we discovered that overexpression of ANKRD1 aggravated intracellular lipid peroxidation and further elevated ROS levels in response to H_2_O_2_. Conversely, these alterations in HK‐2 cells suffered from H_2_O_2_ stimulation can be attenuated by ANKRD1 knockdown (Figure 4D‒G). Consistently, in the mouse model of IRI‐AKI, we also observed that knockdown of ANKRD1 in the I/R‐treated kidneys rescued the anti‐ferroptosis pathway (Figure 4H), reduced lipid metabolite MDA (Figure 4I), and considerably alleviated GSH depletion (Figure 4J). Inhibition of ANKRD1 can mitigate these dysregulations, while high levels of ANKRD1 may exacerbate the aforementioned changes.

### 3.4 ANKRD1 interacts with ACSL3 in renal IRI through the ANK repeat region

**FIGURE 5:**
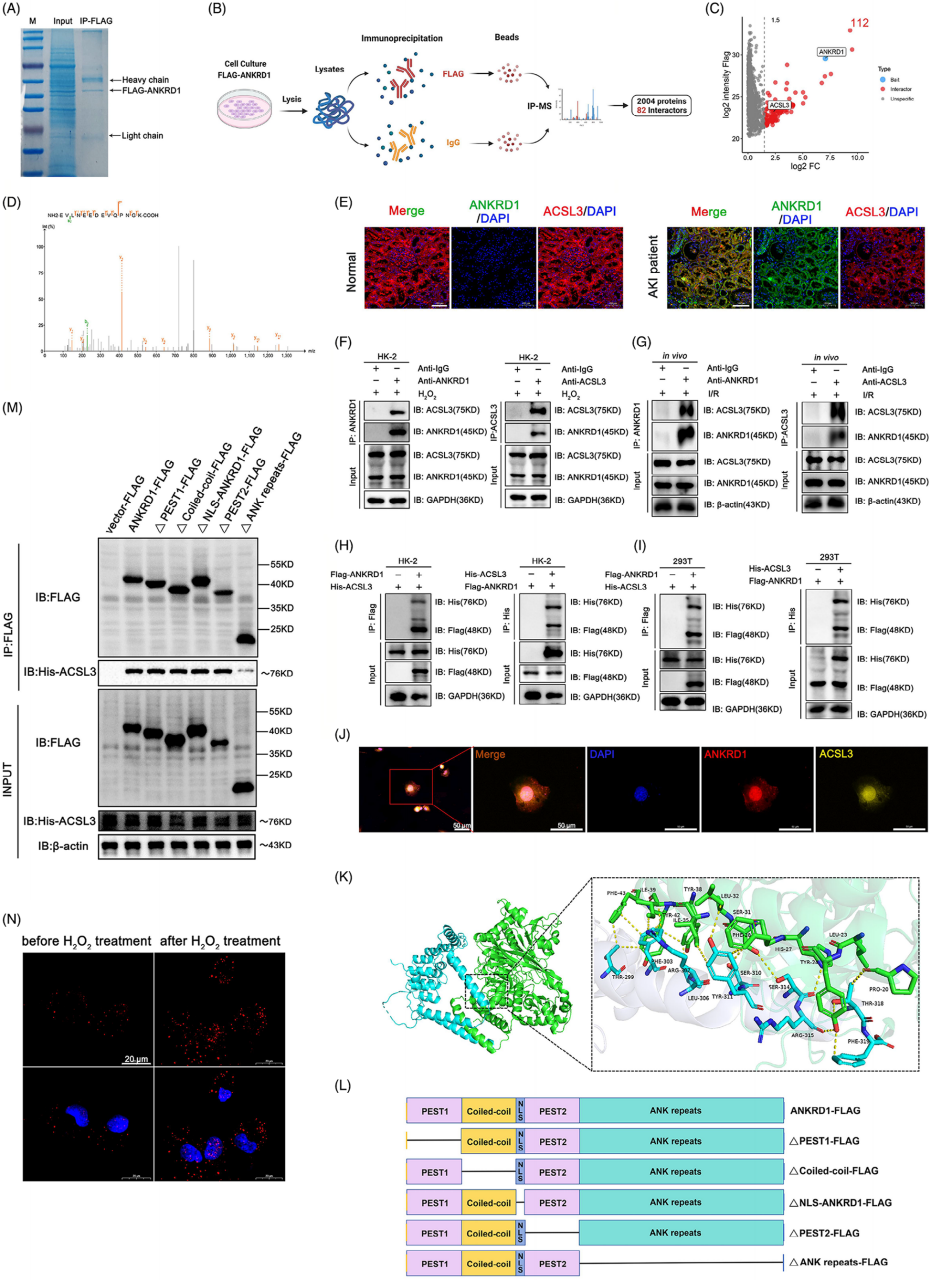
(A) Ankyrin repeat domain 1 (ANKRD1) interacts with acyl‐coenzyme A synthetase long‐chain family member 3 (ACSL3) in renal tubular epithelial cell. Coomassie Blue‐stained gel showing immunoprecipitation (IP) product. (B) Schematic diagram of immunoprecipitation‐mass spectrometry (IP‐MS) procedure. Created with Biorender.com. (C) The volcano plot showing ACSL3 is a potential target protein of ANKRD1. (D) MS/MS spectra of the peptide ‘EVLNEEDEVQPNGK’. Peaks with colour are the detected b ions (green) and y ions (red). (E) Co‐localisation of ANKRD1 and ACSL3 in human renal biopsy specimens. (F) H_2_O_2_‐treated HK‐2 cell lysates were subjected to IP with IgG, ANKRD1 and ACSL3 antibody, respectively, followed by ANKRD1 and ACSL3 immunoblotting. (G) Ischaemia/reperfusion (I/R)‐treated renal tissue lysates were immunoprecipitated with IP with IgG, ANKRD1 and ACSL3 antibody, respectively, followed by ANKRD1 and ACSL3 immunoblotting. (H and I) Representative co‐immunoprecipitation (Co‐IP) images of ANKRD1 and ACSL3 interaction in HK‐2 and HEK 293T cells. The FLAG‐ANKRD1 and His‐ACSL3 plasmids were co‐transfected into cells. (J) Co‐localisation of ANKRD1 and ACSL3 in the nucleus and cytoplasm of HK‐2 cell as determined by IF staining. Scale bar, 50 µm. (K) Interactions of ANKRD1 with ACSL3 as predicted by molecular docking. ACSL3 was selected as the receptor protein and ANKRD1 was selected as the ligand protein. Hydrogen bonds are indicated in yellow. (L) The schematic diagram of human ANKRD1 and its truncated mutants. (M) IP and Western blot analysis of the His‐tagged ACSL3/FLAG‐tagged mutant ANKRD1 proteins interaction in the HK‐2 cells. (N) Detection of ANKRD1 and ACSL3 interaction in HK‐2 cells by proximity ligation assay (PLA). Scale bar, 20 µm.

Given previous findings that ANKRD1 activates P53 in other contexts, and the established role of P53 in modulating SLC7A11 to affect ferroptosis, we initially explored the P53/SLC7A11 pathway. We manipulated ANKRD1 levels in mouse kidneys and RPTCs using rAAV9 and lentivirus, respectively. However, Western blot analysis revealed that altering ANKRD1 expression did not significantly influence P53/SLC7A11 levels in both in vitro and in vivo IRI models (Figure S1). To identify potential binding proteins for ANKRD1, whole‐cell lysates were immunoprecipitated using FLAG primary antibodies. Protein bands were visualised through Coomassie blue staining (Figure 5A), and the resulting immunoprecipitation products were subjected to mass spectrometry analysis (IP‐MS). A total of 82 interacting proteins (cutoff = 4) were identified (Figure 5B). Our discovery of a relationship between ANKRD1 and ferroptosis drew our attention to the reciprocal protein ACSL3 (Figure 5C,D). Dual IF staining showed that in the kidneys of AKI patients, the expression of ACSL3 was decreased in contrast to a significant increase in ANKRD1. There was a significant co‐localisation of ANKRD1 and ACSL3, suggesting that the two proteins interacted with each other (Figure 5E). To validate the IP‐MS results, co‐immunoprecipitation (Co‐IP) analysis was performed. ANKRD1 and ACSL3 were separately immunoprecipitated from 500 µM H_2_O_2_ or H/R‐stimulated HK‐2 cells, and the reciprocal proteins were detected through immunoblotting. ANKRD1 precipitated ACSL3 in RPTCs, while the control IgG did not. Reverse Co‐IP demonstrated that ACSL3 significantly precipitated ANKRD1 in RPTCs (Figures 5F and S7). In I/R‐treated mice kidneys, ANKRD1 and ACSL3 reciprocally precipitated each other (Figure 5G). Additionally, we conducted Co‐IP analysis with epitope‐tagged proteins in HK‐2 and HEK 293T cells. Flag‐tagged ANKRD1 and His‐tagged ACSL3 were found to co‐precipitate in these two cell types (Figure 5H,I). Molecular docking and truncation mutation experiments were subsequently performed to elucidate the specific interaction mechanisms between ANKRD1 and ACSL3. Molecular docking analysis depicted the binding interaction of ANKRD1 and ACSL3. Docking analysis unveiled that the two proteins interacted primarily through hydrogen bonding, with a docking score of −261.32, confidence score of .9026 and ligand root‐mean‐square deviation (RMSD) of 49.21. Further analysis showed that the binding sites on ACSL3 (Pro20, Leu23, Tyr24, His27, Phe28, Ser31, Leu32, Ile35, Tyr38, Ile39, Tyr42, Phe43) and ANKRD1 (Thr 299, Phe 303, Leu 306, Arg 307, Ser 310, Tyr 311, Ser 314, Arg 315, Thr 318 and Phe 319) formed a high‐activity binding pockets via hydrogen bonds, which is rich in hydrophobic and aromatic residues (Table S1). Notably, the 299−319 amino acid sequence of ANKRD1 binds more strongly to ACSL3 through hydrogen bonding (Figure 5K). Taken together, these results suggest that ANKRD1, particularly its ANK repeats domain, can interact with the hydrophobic binding pocket of ACSL3, which was confirmed by truncation mutation experiments. Various truncated mutants of ANKRD1 with different lengths were constructed by separately deleting the PEST1, coiled‐coil, NLS, PEST2 and ANK repeats regions (Figure 5L). IP assays showed that deletion of the ANK repeats domain significantly reduced ACSL3 pull‐down, suggesting the domain is crucial for mediating the ANKRD1‒ACSL3 interaction in RPTCs (Figure 5M). Moreover, ANKRD1 and ACSL3 co‐localised in the nucleus and cytoplasm of HK‐2 cells (Figure 5J). Proximity ligation assay (PLA) assay demonstrated that H_2_O_2_ treatment robustly promoted the ANKRD1‒ACSL3 interaction in HK‐2 cells (Figure 5N). These data indicate the existence of an interaction between ANKRD1 and ACSL3 during renal IRI and H_2_O_2_ and H/R treatment.

### 3.5 ANKRD1 negatively regulates ACSL3 levels to exacerbate ferroptosis in renal IRI

**FIGURE 6:**
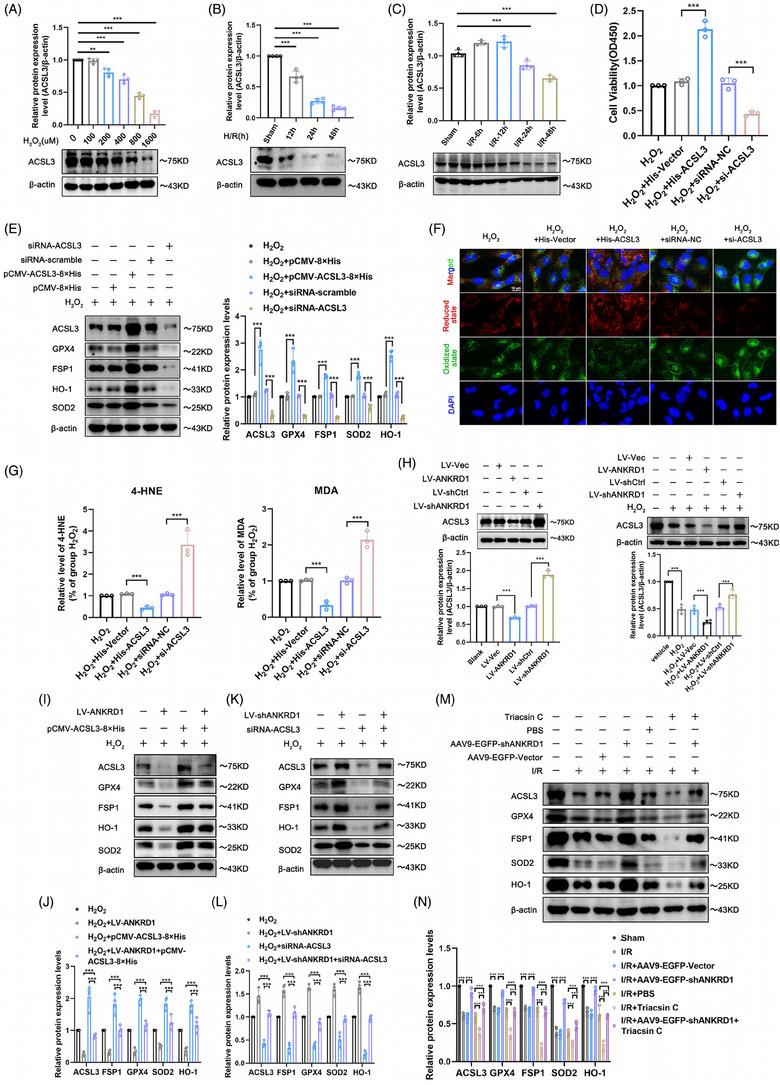
Ankyrin repeat domain 1 (ANKRD1) decreases acyl‐coenzyme A synthetase long‐chain family member 3 (ACSL3) expression and activity, aggravating ferroptosis in renal ischaemia‒reperfusion injury (IRI). (A) Representative immunoblot images and quantification of ACSL3 in H_2_O_2_‐treated HK‐2 cells. (B) Representative immunoblot images and quantification of ACSL3 in H/R‐treated HK‐2 cells. (C) Representative immunoblot images and quantification of ACSL3 in ischaemia/reperfusion (I/R)‐treated mice kidney. (D) Detection of HK‐2 cell viability by cell counting kit‐8 (CCK‐8) assay upon overexpression or knockdown of ACSL3. (E) Representative immunoblot images and quantification of ACSL3, GPX4, FSP1, HO‐1 and SOD2 in H_2_O_2_‐treated HK‐2 cells after overexpression or knockdown of ACSL3. (F) Detection of lipid reactive oxygen species (ROS) by C11 BODIPY assay upon overexpression or knockdown of ACSL3 in HK‐2 cells. (G) Detection of 4‐Hydroxynonenal (4‐HNE) and malondialdehyde (MDA) in HK‐2 cells after intervention with ACSL3. (H) Effects of overexpression or knockdown of ANKRD1 on ACSL3 in HK‐2 cells as determined by Western blot. (I and J) Representative immunoblot images and quantification of ACSL3, GPX4, FSP1, HO‐1 and SOD2 in H_2_O_2_‐treated HK‐2 cells after overexpression of ANKRD1 and ACSL3. (K and L) Representative immunoblot images and quantification of ACSL3, GPX4, FSP1, HO‐1 and SOD2 in H_2_O_2_‐treated HK‐2 cells after knockdown of ANKRD1 and ACSL3. (M and N) Representative immunoblot images and quantification of ACSL3, GPX4, FSP1, HO‐1 and SOD2 in I/R‐treated mice kidney after knockdown of ANKRD1 and ACSL3 by pharmacological inhibition and gene intervention. The data in (A‒E), (G), (I) and (K) are expressed as the mean ± standard deviation (SD). ^**^ *p* < .01; ^***^ *p* < .001.

Distinct from ACSL4, ACSL3 activates MUFA and enhances cellular resistance to ferroptosis.^13^ To date, the involvement of ACSL3 in the pathophysiological process of AKI remains unclear. Therefore, we investigated the role of ACSL3 in renal IRI. First, a substantial reduction in ACSL3 levels was noted in two classical in vitro models of renal IRI (H/R and H_2_O_2_ stimulation) (Figure 6A,B). Second, we consistently observed a reduced ACSL3 level in I/R‐treated mice kidneys (Figure 6C). Notable, in the mouse model of IRI‐AKI, ACSL3 levels exhibited a slight elevation at early time points (12 h post‐I/R, although the difference is not significant), which was different from the observation from in vitro models of renal IRI (H/R stimulation). One possible explanation: the observed discrepancy may stem from the intricate systemic responses that are characteristic of living organisms. Biological systems inherently possess protective and compensatory mechanisms that respond dynamically to disease initiation and progression. In contrast, the isolated cell models tend to be more static and sensitive to exogenous stressors. This disparity highlights a fundamental principle of biology, where cellular responses in vitro may not be directly equate to those in more complex in vivo environments.

CCK‐8 assays showed that high levels of ACSL3 rescued HK‐2 cell viability under H_2_O_2_ treatment, whereas knockdown of ACSL3 decreased HK‐2 cell viability (Figure 6D). Using gain‐of‐function and loss‐of‐function strategies, we explored the role of ACSL3 in ferroptosis in response to H_2_O_2_ and H/R stimulation. ACSL3 knockdown reduced the expression of GPX4, FSP1, SOD2 and HO‐1 in HK‐2 cells, whereas ACSL3 overexpression significantly rescued the activity of these anti‐ferroptosis proteins, which indicated that ACSL3 functioned as an inhibitor of ferroptosis and ACSL3 loss weakened cellular resistance to ferroptosis in H_2_O_2_ and H/R‐induced HK‐2 cells (Figures 6E and S8A). High levels of ACSL3 significantly attenuated the degree of lipid peroxidation and the accumulation of its products, MDA and 4‐HNE. Conversely, ACSL3 deficiency led to the buildup of MDA and 4‐HNE, and exacerbated lipid peroxidation and ferroptosis (Figures 6F,G and S8B,C). Notably, ACSL3 expression in kidney and RPTCs can be regulated by ANKRD1. Intravenous injection of rAAV9 to knockdown ANKRD1 in mice kidneys alleviated I/R‐induced depletion of ACSL3 and GPX4 in the renal cortex (Figure S4). Compared with the Lv‐mock vector + H_2_O_2_/Lv‐mock vector + H/R group, ACSL3 levels decreased in the Lv‐ANKRD1 + H_2_O_2_/Lv‐ANKRD1 + H/R group; however, ANKRD1 knockdown reverted ACSL3 levels. This inhibitory effect of ACSL3 by ANKRD1 was also detected in untreated HK‐2 cells, but was weaker than that in H_2_O_2_ or H/R‐stimulated HK‐2 cells (Figures 6H and S8D,E). Subsequently, rescue experiments in vivo and in vitro were conducted. In H_2_O_2_ and H/R‐treated HK‐2 cells, overexpression of ANKRD1 worsened the depletion of anti‐ferroptosis proteins, while upregulation of ACSL3 restored the activity of these proteins (Figures 6I,J and S8F). Inhibition of ACSL3 blocked the increase in the levels of anti‐ferroptosis proteins resulting from ANKRD1 knockdown (Figures 6K,L and S8G). As expected, ACSL3 and anti‐ferroptosis protein levels in mice kidneys notably were restored following ANKRD1 knockdown. Administering the specific inhibitor Triacsin C to suppress ACSL3 levels in mice further exacerbated the consumption of anti‐ferroptosis proteins compared to the I/R + PBS group, clarifying the protective role of ACSL3 against ferroptosis in vivo. Importantly, the restoration of anti‐ferroptosis proteins brought about by ANKRD1 knockdown was significantly reversed by ACSL3 inhibitor Triacsin C, suggesting that the regulatory role of ANKRD1 on ferroptosis in renal IRI depends on ACSL3 (Figure 6M,N). ACSL1 and ACSL4 are the core enzymes regulating PUFA, and dysregulation of their expression can also exacerbate ferroptosis. It remains unknown whether ANKRD1 regulates ACSL1/4. Thus, the expression of ANKRD1 was interfered in vivo and in vitro to observe its effects on ACSL1/4. ACSL1/4 levels increased in renal IRI, aligning with expectation. However, I/R‐induced elevation of ACSL1/4 was not inhibited by AAV9‐mediated knockdown of ANKRD1 in mouse model (Figure S2A). Furthermore, neither overexpression nor knockdown of ANKRD1 in RPTCs influenced ACSL1/4 activity, despite the alterations in ANKRD1 levels (Figure S2B,C). Collectively, our in vivo and in vitro findings suggest that ANKRD1 is unlikely to regulate ACSL1/4 in the context of renal IRI. These findings buttress that ANKRD1 directly interacts with ACSL3 and negatively regulates ACSL3 levels to exacerbate ferroptosis in renal IRI.

### 3.6 ANKRD1 promotes ACSL3 degradation via the ubiquitin‒proteasome pathway

**FIGURE 7:**
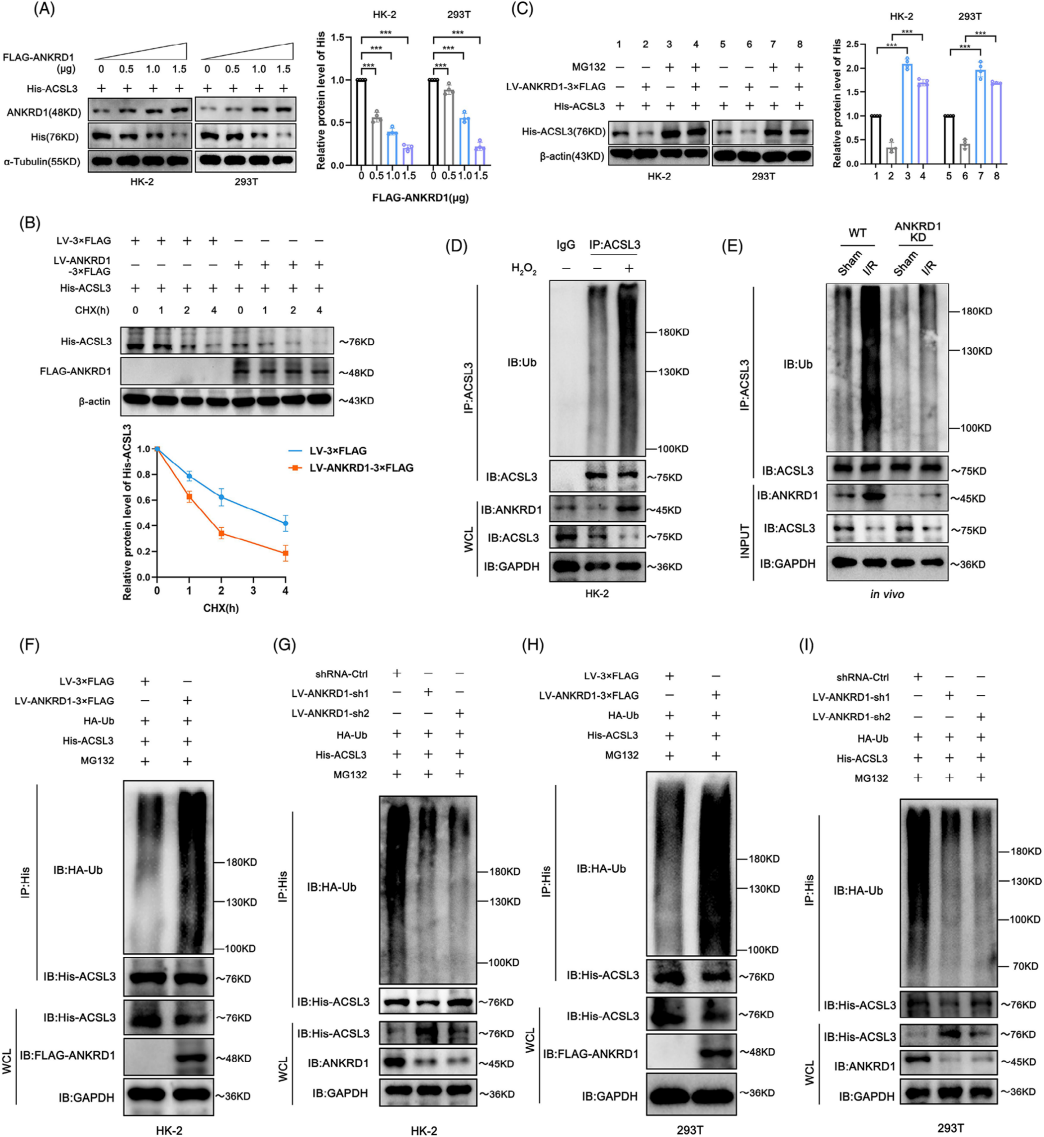
Ankyrin repeat domain 1 (ANKRD1) promotes acyl‐coenzyme A synthetase long‐chain family member 3 (ACSL3) degradation via the ubiquitin‒proteasome pathway. (A) Immunoblot analysis and quantification of His levels after transfected with the indicated doses of FLAG‐ANKRD1 plasmids in HK‐2 and HEK 293T cells. (B) ANKRD1 overexpression reduced the stability of ACSL3. HK‐2 cells were treated with cycloheximide (CHX) for the indicated time. (C) HK‐2 and HEK 293T cells were treated with MG132. The levels of ACSL3 were detected by Western blot analysis. (D) Detection of endogenous ubiquitination levels of ACSL3 in H_2_O_2_‐treated HK‐2 cells. (E) Detection of endogenous ubiquitination levels of ACSL3 in ANKRD1‐deficient mice kidney. (F‒I) HK‐2 and HEK 293T cells were treated with 5 µM MG132 for 5 h prior to harvest. Cell lysate was immunoprecipitated with His‐tag antibody and immunoblotted as indicated. The data in (A‒C) are expressed as the mean ± standard deviation (SD). ^***^ *p* < .001.

In HK‐2 and HEK 293T cells, increasing ANKRD1 expression effectively lowered ACSL3 protein levels in a dose‐dependent manner (Figure 7A). Given that there exists a direct interaction between ANKRD1 and ACSL3, we hypothesised that ANKRD1 could regulate ACSL3 activity at the post‐translational level. To test it, we administered cycloheximide to HK‐2 cells to inhibit translation and observed rapid degradation of ACSL3 protein in the ANKRD1 overexpression group, while the control group showed an extended half‐life of ACSL3 (Figure 7B). Moreover, when HK‐2 and HEK 293T cells were treated with the proteasome inhibitor MG132, the decline in ACSL3 levels caused by ANKRD1 overexpression was reversed (Figure 7C). These findings indicate that ANKRD1‐mediated degradation of ACSL3 involves the ubiquitination pathway at the post‐translational level. To validate this point, we further examined the endogenous ubiquitination levels in the in vitro/in vivo models of renal IRI. We observed a significant increase in ACSL3 ubiquitination levels in H_2_O_2_ and H/R‐stimulated HK‐2 cells compared to controls (Figures 7D and S9A). Similarly, in the mouse model of IRI‐AKI, ACSL3 ubiquitination levels in I/R‐treated kidneys also showed a remarkable elevation, which could be suppressed by ANKRD1 knockdown (Figure 7E). Furthermore, we found that overexpression or knockdown of ANKRD1 in HK‐2 and HEK 293T cells alone was sufficient to enhance or repress ACSL3 ubiquitination (Figure 7F‒I). Taking these results together, ANKRD1 promotes the degradation of ACSL3 via ubiquitin‒proteasome pathway.

### 3.7 E3 ligase TRIM25 is responsible for ANKRD1‐mediated ubiquitination of ACSL3

**FIGURE 8:**
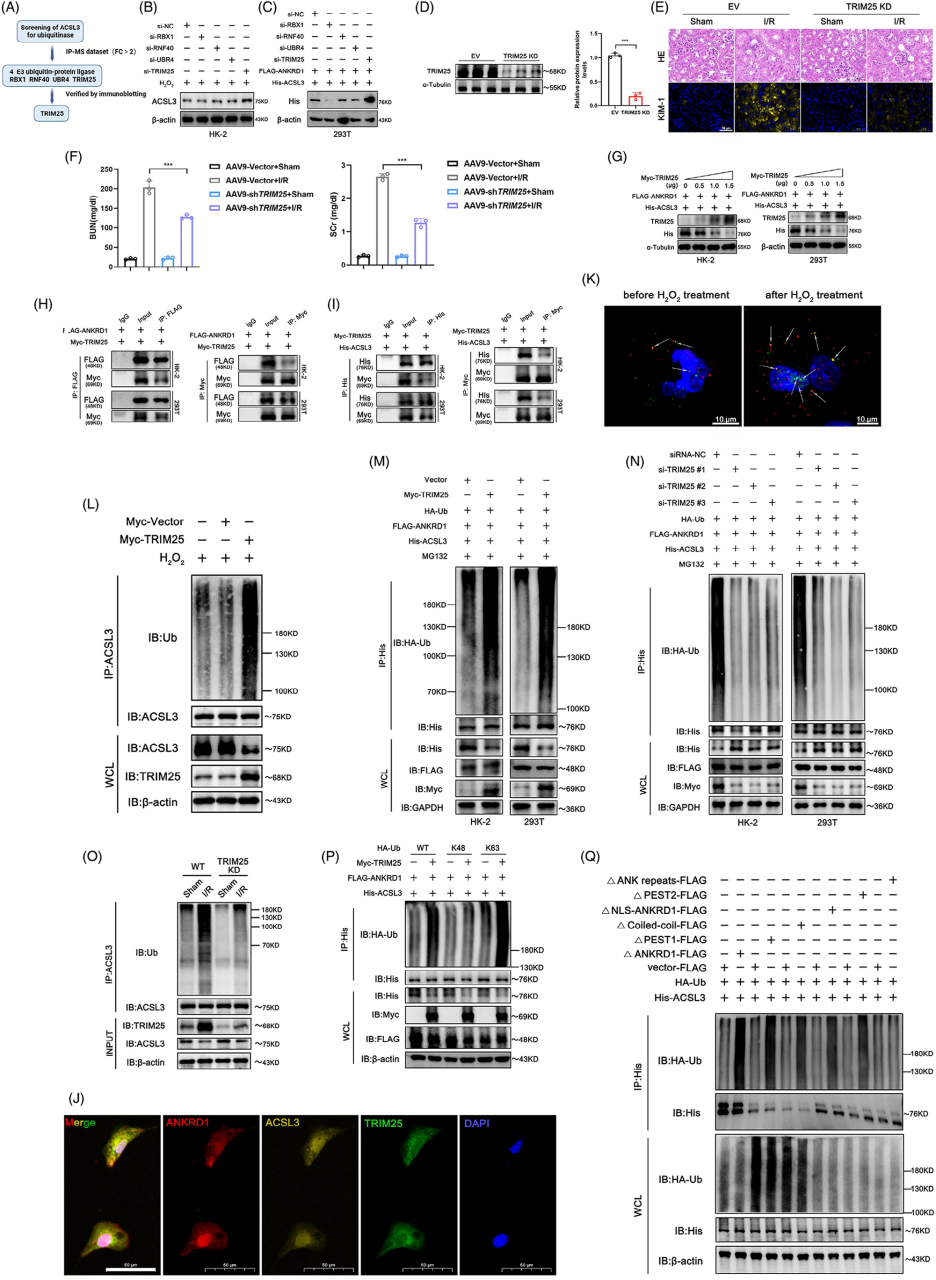
A) Ankyrin repeat domain 1 (ANKRD1) facilitates acyl‐coenzyme A synthetase long‐chain family member 3 (ACSL3) degradation by collaborating with the E3 ligase tripartite motif containing 25 (TRIM25) to catalyse the K63‐linked polyubiquitination of ACSL3. Identification of possible E3 ligases that regulate the stability of ACSL3 and interact with ANKRD1 simultaneously. (B) Representative immunoblot images of ACSL3 in H_2_O_2_‐treated HK‐2 cells after knockdown of the indicated genes. (C) Immunoblot images of ACSL3 in HEK 293T cells after ANKRD1 overexpression and knockdown of the indicated genes. (D) Immunoblotting of renal cortical tissues confirmed the knockdown efficiency of TRIM25 protein. (E) Renal tissues were collected for haematoxylin and eosin (HE) staining and IF staining of kidney injury molecule 1 (KIM‐1). Scale bar, 50 µm. (F) Serum creatinine (SCr) and blood urea nitrogen (BUN) measurement. (G) Immunoblot analysis of His and TRIM25 levels after transfected with the indicated doses of Myc‐TRIM25 plasmids in HK‐2 and HEK 293T cells. (H) Co‐immunoprecipitation (Co‐IP) assay of TRIM25 and ANKRD1 in HK‐2 and HEK 293T cells. (I) Co‐IP assay of TRIM25 and ACSL3 in HK‐2 and HEK 293T cells. (J) Co‐localisation of ANKRD1, ACSL3 and TRIM25 in HK‐2 cells as determined by IF staining. Scale bar, 50 µm. (K) The interaction between TRIM25, ANKRD1 and ACSL3 was significantly increased in H_2_O_2_‐stimulated HK‐2 cells as determined by proximity ligation assay (PLA) assay. Red: interaction between ANKRD1 and TRIM25; green: interaction between ACSL3 and TRIM25. Scale bar, 10 µm. (L) High levels of TRIM25 were observed to augment endogenous ubiquitination of ACSL3 in H_2_O_2_‐stimulated HK‐2 cells. Myc‐tagged TRIM25 was transfected into HK‐2 cells, and endogenous ubiquitination levels of ACSL3 were detected by immunoprecipitation and Western blot analysis. (M and N) HK‐2 and HEK 293T cells were treated with 5 µM MG132 for 5 h prior to harvest. Cell lysate was immunoprecipitated with His‐tag antibody and immunoblotted as indicated. (O) Detection of endogenous ubiquitination levels of ACSL3 in TRIM25‐deficient mice kidney. (P) HK‐2 cells were co‐transfected with indicated plasmids, and ACSL3 ubiquitination was analysed. (Q) ANK repeat domain is required for ANKRD1‐mediated ubiquitination of ACSL3 in HK‐2 cells. The PEST1, coiled‐coil, NLS, PEST2 and ANK repeats regions were deleted separately to construct various ANKRD1 truncation. HK‐2 cells were transfected with FLAG‐tagged wild‐type (WT) or mutant plasmids of ANKRD1, HA‐Ub and His‐ACSL3. Cell lysate was immunoprecipitated with His‐tag antibody and immunoblotted as indicated. ^***^ *p* < .001.

ANKRD1 promotes ACSL3 degradation via the ubiquitin‒proteasome pathway, but it lacks an E3 ligase activity. Therefore, we searched our IP‐MS data to identify the potential E3 ligases that could modulate ACSL3 stability and directly interact with ANKRD1 (Figure 8A). As a result, four candidate proteins were identified: RBX1, RNF40, UBR4 and TRIM25. After knockdown of each of these four E3 ligases, only TRIM25 silencing restored endogenous ACSL3 levels in H_2_O_2_ and H/R‐stimulated HK‐2 cells (Figures 8B and S9B). When both inhibition of TRIM25 and overexpression of ANKRD1 concurred in HEK 293T cells, ACSL3 protein levels significantly increased (Figure 8C). TRIM25 was selected for further study. The function of TRIM25 in renal IRI was explored in vivo experiments. We utilised rAAV9‐shRNA to interfere with TRIM25 levels in mouse kidney, and the knockdown efficiency of TRIM25 was verified by Western blot (Figure 8D). TRIM25 knockdown effectively reduced early pathological damage of kidney, such as vacuolar degeneration and cellular flattening along with a decrease in KIM‐1 (Figure 8E). Notably, TRIM25 knockdown markedly improved renal function in mice (Figure 8F). Similar to ANKRD1, TRIM25 lowered ACSL3 protein expression considerably in a dose‐dependent manner (Figure 8G). Co‐IP data indicated that the amount of ANKRD1 and ACSL3 bound to the same quantity of TRIM25 in the I/R group was significantly higher compared to the sham group, implying that renal IRI leads to an increase in the endogenous binding of ANKRD1 and ACSL3 with TRIM25 (Figure S9C). The interactions of exogenous TRIM25 with ANKRD1 and ACSL3 were also validated in vitro (Figure 8H,I). Subcellular co‐localisation of these proteins in HK‐2 cells also further buttresses their functional relationship (Figures S10 and 8J). To better illustrate the dynamic interactions among TRIM25, ANKRD1 and ACSL3, PLA assay was performed. In untreated HK‐2 cells, positive interaction signals were observed between TRIM25 and ANKRD1/ACSL3, as evidenced by the overlap of red and green positive signals, indicating baseline binding among the three proteins in a basal condition. Upon H_2_O_2_ treatment, both red and green signals within the cells underwent a notable intensification, accompanied by a substantial augmentation in their overlap, indicating an enhanced interaction among TRIM25, ANKRD1 and ACSL3 (Figure 8K). High levels of TRIM25 were observed to augment endogenous ubiquitination of ACSL3 in H_2_O_2_ and H/R‐stimulated HK‐2 cells (Figures 8L and S9D). Under ANKRD1 over‐expression circumstances, which mimic one of the primary molecular phenotypes of renal IRI, higher TRIM25 levels enhanced ACSL3 ubiquitination, whereas TRIM25 knockdown inhibited this process (Figure 8M,N). Notably, I/R‐induced increased ubiquitination of ACSL3 was significantly ameliorated after knockdown of TRIM25 in vivo, confirming the crucial regulatory role of TRIM25 on ACSL3 through ubiquitination modification (Figure 8O). Moreover, K48 and K63 linkages are the main chains involved in ubiquitin‒proteasome pathway. TRIM25 has been reported to mediate ubiquitination through K48 and K63 linkages.^15^, ^28^, ^29^ Our data revealed that under conditions of ANKRD1 overexpression, TRIM25 promoted K63‐linked polyubiquitination of ACSL3, but had no significant effects on K48‐linked polyubiquitination (Figure 8P). To identify the domain required for ANKRD1‐mediated ubiquitination of ACSL3, truncated mutants of ANKRD1 with different lengths were constructed. Deletion of the ANK repeat domain resulted in a notable attenuation of the ANKRD1‐mediated increased ubiquitination of ACSL3 compared to other mutation schemes (Figure 8Q). Notably, interfering with ANKRD1 levels in vivo and in vitro has minimal impact on the transcription levels of ACSL3 and TRIM25, thereby ruling out the possibility of ANKRD1 functioning as a transcriptional cofactor to regulate ACSL3 and TRIM25 (Figure S11). These findings highlight the interplay pattern between TRIM25, the newly discovered dose‐dependent negative modulator of ACSL3 in renal IRI, and ANKRD1 in regulating ACSL3 activity.

### 3.8 Damage effects on RPTCs mediated by ANKRD1 are directly associated with the function of TRIM25

**FIGURE 9:**
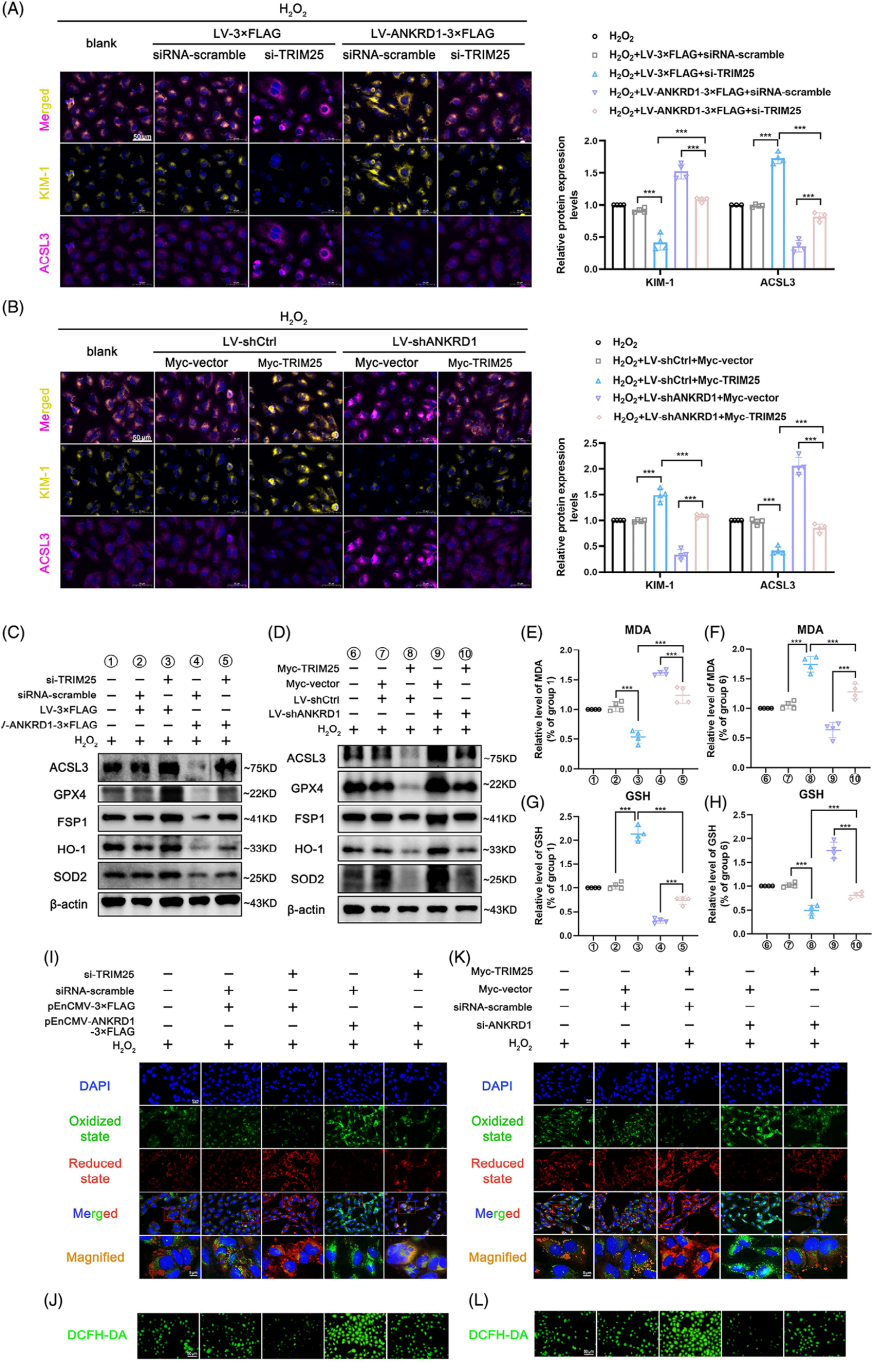
Ankyrin repeat domain 1 (ANKRD1)‐mediated cell damage are correlated with the function of tripartite motif containing 25 (TRIM25). (A and B) Representative cellular images and quantification of acyl‐coenzyme A synthetase long‐chain family member 3 (ACSL3) and kidney injury molecule 1 (KIM‐1) IF staining. Scale bar, 50 µm. (C and D) Representative immunoblot images of ACSL3, GPX4, FSP1, HO‐1 and SOD2 in HK‐2 cells with different treatments. (E and F) Malondialdehyde (MDA) levels in HK‐2 cells with different treatments. (G and H) Glutathione (GSH) levels in HK‐2 cells with different treatments. (I‒L) Representative images of intracellular lipid peroxidation and total reactive oxygen species (ROS) in HK‐2 cells with different treatments. The data in (A), (B) and (E‒H) are expressed as the mean ± standard deviation (SD). ^***^ *p* < .001.

Next, we questioned whether the damage effects mediated by ANKRD1 are directly associated with the function of TRIM25. Our findings demonstrated that silencing TRIM25 reduced KIM‐1 expression in HK‐2 cells treated with H_2_O_2_, while elevating ACSL3 levels. Furthermore, knockdown of TRIM25 prevented cell injury induced by ANKRD1 overexpression, whereas the protective effect resulting from ANKRD1 knockdown could be weakened by TRIM25 overexpression (Figure 9A,B). In HK‐2 cells, TRIM25 knockdown effectively blocked the reduction of anti‐ferroptosis proteins, MDA accumulation, GSH depletion, aggravation of lipid peroxidation, and enhanced intracellular ROS induced by ANKRD1 overexpression (Figures 9C‒J and S12). Conversely, TRIM25 overexpression weakened the anti‐ferroptosis effect caused by ANKRD1 knockdown (Figure 9C‒J).

## 4 DISCUSSION

**FIGURE 10:**
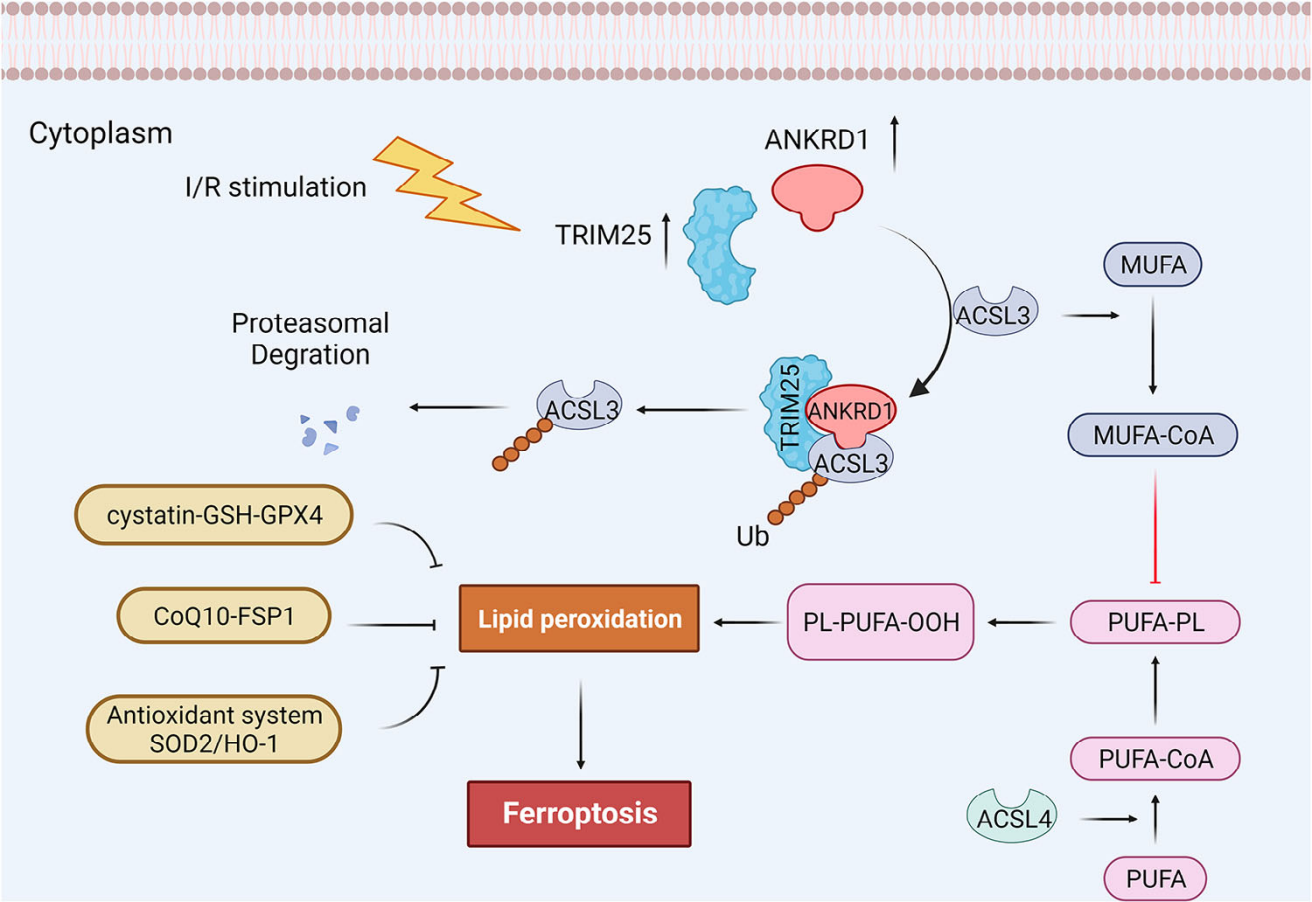
Schematic of signalling pathways related to the effects of ankyrin repeat domain 1 (ANKRD1)/tripartite motif containing 25 (TRIM25)/acyl‐coenzyme A synthetase long‐chain family member 3 (ACSL3) in ischaemia/reperfusion (I/R)‐induced renal injury. Created with Biorender.com.

Renal IRI is widely recognised as a significant contributor to AKI due to the kidney's high susceptibility to IRI.^11^ In clinical practice, renal IRI commonly occurs during kidney transplantation, partial nephrectomy and cardiac surgery.^30^, ^31^, ^32^ The combination of renal tissue ischaemia‐induced hypoxia and subsequent reoxygenation upon blood flow restoration leads to progressive renal tissue damage and renal tubular cell death.^33^, ^34^ Initially, apoptosis and necrosis were identified as the predominant form of cell death in kidney IRI.^35^ According to latest studies, lipid peroxidation‐dependent ferroptosis is also a dominant driver of renal IRI, challenging the conventional understanding of renal cell death.^35^, ^36^, ^37^, ^38^ Nevertheless, the mechanism is still obscure. In the present work, we have demonstrated: (1) ANKRD1, which minimally express in normal kidneys, is rapidly activated in I/R‐stimulated RPTCs in vivo and in vitro; (2) ANKRD1 directly interacts with ACSL3 to facilitate its degradation, thereby enhancing lipid peroxidation and ferroptosis in RPTCs; (3) ANKRD1 promotes K63‐linked polyubiquitination of ACSL3 by synergizing with the E3 enzyme TRIM25; (4) ANKRD1 repression effectively mitigates kidney damage and preserves renal function (Figure  10).

Human *ANKRD1*, located on human chromosome 10q23.31, is highly conserved among mammals and encodes a 319‐amino‐acid protein.^39^, ^40^, ^41^ The ANKRD1 possesses several distinct domains or motifs, including a coiled‐coil domain, tandem ankyrin repeats, nuclear localisation signature motifs, PEST protein degradation sequences and probable post‐translational modification sites.^8^ Among these components, the ankyrin repeat units, composed of a 30‐32‐residue motif, are known to mediate protein‒protein interactions.^42^ The ANKRD1 protein is subcellular localised in the cell nucleus and cytoplasm, and its two potential nuclear localisation signals imply that it can be actively transported into the nucleus.^43^ Initially discovered to be a nuclear transcriptional co‐factor, ANKRD1 was shown to adversely regulate the expression of several genes unique to the heart, including MLC‐2v, cardiac troponin C and atrial natriuretic factor.^39^, ^44^ Numerous studies suggested that ANKRD1 is mainly involved in disease progression and adaptive responses of cardiac hypertrophy, dilated cardiomyopathy and cardiac fibrosis.^8^, ^45^, ^46^ Recent evidence uncovered the multifunctionality of ANKRD1. In ovariectomised mice, ANKRD1 accelerates osteogenic differentiation and bone formation via stimulating Wnt pathway.^7^ The global deletion of ANKRD1 exacerbates dermal fibroblast dysfunction, resulting in extensive necrosis of ischaemic skin flaps and delayed excisional wound healing.^9^ ANKRD1 is also implicated in kidney disease. It is intensely induced in the podocytes of glomerulonephritis patients but barely detectable in those of normal controls.^47^ Interestingly, the level of ANKRD1 protein in podocytes of patients with lupus nephritis correlates with the severity of proteinuria.^47^ In mouse kidneys with unilateral ureter obstructive nephropathy, the mRNA abundance of ANKRD1 is significantly elevated.^48^ Our research firstly presents the proof that ANKRD1 regulates renal IRI. ANKRD1 is barely expressed in normal kidneys, however, our RNA‐seq data and bioinformatics revealed a substantial increase in ANKRD1 mRNA abundance in I/R‐treated kidneys. High levels of ANKRD1 protein were also detected in I/R‐treated renal tissues, particularly in RPTCs. ANKRD1 levels were markedly upregulated as the severity of injury increased in vivo and in vitro. Knockdown of ANKRD1 in the kidney reduced renal tissue damage and restored renal function. Consistently, the damaging effects of ANKRD1 were validated in vitro.

Ferroptosis alters the progression of AKI and impedes recuperation in addition to serving as a pathogenesis mechanism for renal damage.^36^ It has been demonstrated that AKI patients with unrecovered renal function (defined as continuous dialysis requirement) have higher levels of oxidised phosphatidylethanolamine (PE) (a sign of ferroptosis) compared to recovered patients.^49^ Repressing ferroptosis has shown encouraging therapeutic benefits in kidney diseases, especially in AKI. Ferroptosis‐specific inhibitors can increase renal proximal tubular plasticity and alleviate inflammation so as to promote kidney repair.^50^ Recently, Zhao et al. found that ANKRD1 was the most significantly upregulated DEG in HK‐2 cells subjected to calcium oxalate crystal stress. They also discovered that ANKRD1 levels in the CaOx‐crystal‐induced HK‐2 cells correlated positively with P53 levels and negatively with SLC7A11 levels. However, they did not further investigate this link in an animal kidney stone model or determine whether and how ANKRD1 directly or indirectly affects P53 and SLC7A11 levels. In fact, we examined P53 levels in IRI‐induced AKI, unfortunately no distinct differences were observed. Perhaps this observed discrepancy stems from differences in the pathophysiological conditions between urolithiasis and renal IRI.^10^ The study we conducted provided proof that, during the acute stage of kidney IRI, ANKRD1 aggravated ferroptosis. H_2_O_2_ and H/R‐stimulated HK‐2 cells exhibited characteristic alterations associated with ferroptosis, including rapidly increased intracellular lipid peroxidation and total ROS, and collapsed antioxidant system, as evidenced primarily by GSH depletion and notable consumption of the two major anti‐ferroptosis members, GPX4 and FSP1. ANKRD1 knockdown in HK‐2 cells potently alleviated the aforementioned changes, while overexpression of ANKRD1 further intensified the extent of renal ferroptosis. These observations further were buttressed by our evidence that ANKRD1 knockdown alone prior to I/R treatment in mouse kidneys was sufficient to effectively restrain the extent of renal ferroptosis.

Iron‐dependent peroxidation of membrane phospholipids and subsequent plasma membrane disruption are hallmark events of ferroptosis.^51^, ^52^ Ferroptosis can be triggered by a decrease in cellular reducing capacity.^51^ The initiation of ferroptosis commences once other protective mechanisms fail to restrain the accumulation of excessive toxic byproducts derived from the peroxidation of specific phospholipids, particularly PE.^36^ Lipid peroxidation is a free radical‐driven reaction and metabolises PUFA in cell membranes to lipid hydroperoxides.^13^ MUFA and PUFA exhibit contrasting roles in ferroptosis due to their distinct susceptibilities to oxidation.^53^ The ACSL family converts free long‐chain fatty acids to fatty acyl‐coenzyme A esters and is involved in both anabolic and catabolic processes.^13^ ACSL4 preferentially catalyses 20‐carbon PUFA substrates such as arachidonic acid (AA) and adrenergic acid (ADA), accelerating their conversion to AA‐CoA and ADA‐CoA to generate lipid peroxides.^13^, ^54^ In contrast, ACSL3 is required to activate MUFA, which modifies the characteristics of the cell membrane by substituting PUFA, thereby reducing the oxidative sensitivity of plasma membrane lipids in a few hours.^13^ Accumulating evidence suggested ACSL3 activity is strongly implicated in ferroptosis susceptibility. Exogenous MUFAs reduces the incorporation of PUFA into phospholipids and promotes a state of cell resistance to ferroptosis in an ACSL3‐dependent manner.^55^ ACSL3 also regulates the accumulation of lipid droplets in clear cell renal cell carcinoma (ccRCC) and modulates the sensitivity of ccRCC to ferroptosis in a manner dependent on the composition of exogenous fatty acids.^56^ Methionine adenosyltransferase 2A (MAT2A) is responsible for the generation of S‐adenosylmethionine, which can transactivate ACSL3 by promoting trimethylation of lysine‐4 on histone H3 at its promoter region, leading to ferroptosis resistance.^57^ Furthermore, the natural bioactive compound baicalin can alleviate ferroptotic injury by elevating ACSL3 levels, thereby ameliorating cerebral IRI.^58^ In order to investigate how ANKRD1 modulates ferroptosis in renal IRI, we performed IP‐MS to identify ACSL3, rather than ACSL1/4, as the interacting protein of interest. Although ACSL1/4 is also implicated in the ferroptosis‐mediated renal IRI, our experimental results excluded the possibility that ANKRD1 regulates ACSL1/4 in renal IRI. Our Co‐IP analysis in vivo and in vitro, PLA assay, and IF staining data validated the interaction between ANKRD1 and ACSL3. The ANK repeat region was identified as the essential domain that mediates the interaction between ANKRD1 and ACSL3. Moreover, high levels of ANKRD1 repressed the activity of ACSL3 in IRI kidneys and H_2_O_2_ or H/R‐stimulated HK‐2 cells. Blockage of ACSL3 in vivo and in vitro by pharmacological inhibition and gene intervention, respectively, attenuated the anti‐ferroptosis effect caused by ANKRD1 inhibition. These findings suggest that ANKRD1 reduces ACSL3 expression to exacerbate ferroptosis in renal IRI.

The ubiquitin‒proteasome system (UPS) selectively degrades most cellular proteins through an ATP‐driven process. Protein ubiquitination, a multi‐enzymatic process, involves the covalent attachment of ubiquitin to a substrate protein, which subsequently enables the proteasome to recognise and degrade the protein.^59^ Perturbation of UPS contributes to the pathogenesis and progression of kidney diseases, such as renal fibrosis, AKI and muscle atrophy associated with chronic kidney disease.^59^, ^63^, ^64^, ^65^ The alterations in E3 ubiquitin ligase expression and activity in the post‐ischaemic renal environment significantly impact the fate of RPTCs.^60^, ^61^, ^62^ ANKRD1 has been demonstrated to regulate protein ubiquitination post‐translationally. ANKRD1 modulates caveolin‐3 (CAV3) expression by lowering ubiquitination levels to activate the Wnt/β‐catenin signalling, thereby ameliorating pathological alterations.^7^ In rat sympathetic neurons, ANKRD1 stabilises tyrosine hydroxylase proteins by inhibiting ubiquitination but not mRNA levels, promoting catecholamine biosynthesis.^66^ Moreover, ANKRD1 can interact with E3‐ubiquitin ligases, MuRF1 and MuRF2, through the coiled‐coil structural domain.^67^ Our data also show that ANKRD1 is able to directly interact with the E3‐ubiquitin ligases TRIM25, contributing to ACSL3 degradation post‐translationally in a dose‐dependent manner. First, endogenous ubiquitination assays revealed that the ubiquitination levels of ACSL3 increased in both the in vivo and in vitro model of renal IRI; in comparison to control, ANKRD1 deficiency substantially decreased the ubiquitination levels of ACLS3 in the mouse kidney. Following this, we attempted to intervene in ANKRD1 expression in vitro to observe the effect on ACSL3 ubiquitination. ANKRD1 overexpression elevated the ubiquitination levels of ACSL3 in HK‐2 and HEK 293T cells, while the opposite effects were observed for ANKRD1 knockdown. Nevertheless, ANKRD1 itself does not have E3 ligase activity, prompting us to explore intermediate mechanisms further. We searched the IP‐MS data for E3 ligases that can interact with ANKRD1, and found that these E3 ligases (RBX1, RNF40, UBR4 and TRIM25) were possibly involved in ACSL3 degradation. Following immunoblotting confirmation, we concentrated on TRIM25. AA9‐mediated knockdown of TRIM25 significantly alleviated renal injury and rescued renal function. According to Co‐IP analysis, PLA assay and IF staining, TRIM25 can interact with both ANKRD1 and ACSL3. In HK‐2 and HEK 293T cells, high levels of TRIM25 promoted ACSL3 ubiquitination in the form of K63‐linked polyubiquitination. The ANK repeat region was also identified as the required domain for ANKRD1‐mediated ubiquitination of ACSL3. Notably, we found that ANKRD1‐mediated cell damage and ferroptosis in RPTCs were strongly associated with the activity of the E3 ligase TRIM25, and TRIM25 knockdown potently alleviated ANKRD1‐induced ACSL3 suppression and negative consequences.

Collectively, this study underscores the pivotal role of the ANKRD1/TRIM25/ACSL3 axis in renal IRI. ANKRD1 was found to be upregulated in both IRI‐AKI mouse models and in vitro models, and inhibiting ANKRD1‐mitigated renal failure in mice and damage of HK‐2 cells. Importantly, the opposing effects of ANKRD1 knockdown and overexpression on cell viability and injury strongly demonstrated ANKRD1's effective role in regulating cellular responses to oxidative stress and ischaemia. One of the key findings of this study is that ANKRD1 contributes to renal IRI by exacerbating ferroptosis. ACSL3, an indispensable key enzyme for the synthesis of intracellular MUFA‐CoA, acts as a barrier to ferroptosis. A crucial breakthrough in our research is the identification of ACSL3 as an interacting partner of ANKRD1 in proximal tubular cells during renal IRI. By binding to ACSL3 and reducing its levels, ANKRD1 promotes lipid peroxidation, a hallmark of ferroptosis. Surprisingly, the downregulation of ACSL3 by ANKRD1 is achieved by influencing ACSL3 ubiquitination, with the ANK Repeats domain being the primary contributor to this function of ANKRD1. Given ANKRD1's lack of ubiquitination catalytic function, it does not act independently with ACSL3, and TRIM25 was identified as a collaborator. TRIM25 is recruited by ANKRD1 and binds to ACSL3 to form a complex that exerts its biological function during IRI, and TRIM25 is proven to catalyse K63‐linked ubiquitination of ACSL3. Interestingly, we observed that TRIM25 silencing significantly attenuated the post‐translational degradation of ACSL3 induced by ANKRD1. Our findings, for the first time, expand the roles played by these proteins to the regulation of ferroptosis in the context of renal IRI, revealing a novel connection between lipid metabolism and oxidative stress‐induced cell death.

As HK‐2 cells are immortalised human renal proximal tubule epithelial cell lines, they may carry genetic alterations that could affect their behavior and response to stimuli. These potential genetic changes could impact our study results. However, based on our observations in this present study, the results obtained from in vitro studies did not significantly differ from those obtained from in vivo studies. After all, our findings are primarily based on in vivo studies, with in vitro studies serving merely as a supplement. In conclusion, utilizing both genetic and pharmacological approaches, we uncovered a unique function for ANKRD1 in kidney IRI, and inhibition of ANKRD1 effectively protected against the kidney injury and preserved renal function. ANKRD1 promoted the K63‐linked polyubiquitination of ACSL3 by modulating TRIM25 to activate the ferroptosis cascade response, which resulted in damage to tubular cells and exacerbating AKI. These findings point to ANKRD1 as a prospective treatment target for renal IRI.

## 5 CONCLUSIONS

ANKRD1 promotes the K63‐linked ubiquitination of ACSL3 by regulating the E3 ligase TRIM25, expediting the post‐translational degradation of ACSL3 and ultimately exacerbating renal IRI.

## AUTHOR CONTRIBUTIONS

All authors contributed to this present work. *Investigation, data acquisition and analysis and manuscript drafting*: Shangting Han. *Cell assay and animal study assistance*: Jiayu Guo and Chenyang Kong. *Manuscript review and revising*: Jun Li. *Data analysis and investigation*: Fangyou Lin, Jiefu Zhu, Tianyu Wang, Qi Chen, Yiting Liu and Haochong Hu. *Supervision, project administration and funding acquisition*: Tao Qiu, Fan Cheng and Jiangqiao Zhou.

## CONFLICT OF INTEREST STATEMENT

The authors declare they have no conflicts of interest.

## CONSENT FOR PUBLICATION

None of the results of this manuscript have been published by us or by any other third party.

## ETHICS STATEMENT

The trial adhered to the Declaration of Helsinki. The research protocol received approval from the Human Subjects Committee, Renmin Hospital of Wuhan University (WDRY2021‐KS059). Informed consent was obtained from all patients. All procedures involving the mice were carried out according to the National Institutes of Health Guide for the Care and Use of Laboratory Animals and authorised by the Ethical Committee for Animal Experimentation, Renmin Hospital of Wuhan University. The local ethics committee, adhering to the principles outlined in the Basel Declaration, approved the study design on 9 March 2023 (approval no. WDRM20230304B).

## Supporting information



Supporting information

Supporting information

## Data Availability

The data that support the findings of this study are available from the corresponding author upon reasonable request.
