# Supplementary material for: ANKRD1 aggravates renal ischaemia‒reperfusion injury via promoting TRIM25‐mediated ubiquitination of ACSL3
Source: Clin Transl Med. 2024 Sep 17;14(9):e70024. doi: 10.1002/ctm2.70024 (PMC11406046; doi:10.1002/ctm2.70024)
Supplement: Supplementary file 1 — Supporting information [file CTM2-14-e70024-s002.pdf]

Supplementary Data

Fig. S1. Altering ANKRD1 expression can not significantly impact P53/SLC7A11 levels in both *in vitro* and *in vivo* IRI models.
A. Representative immunoblot images of P53 and SLC7A11 in mice renal tissues. rAAV9-shRNA was utilized to knockdown ANKRD1 in mouse kidney. **B and C.** Representative immunoblot images of P53 and SLC7A11 in H_2O_2 treated HK-2 cells. Lv-FLAG-ANKRD1 and Lv-shANKRD1 were utilized to overexpress or knockdown ANKRD1 in HK-2 cells respectively. n.s. no significance.

Fig. S2. Altering ANKRD1 expression can not significantly impact ACSL1/4 levels in both *in vitro* and *in vivo* IRI models.
A. Representative immunoblot images of ACSL1 and ACSL4 in mice renal tissues. rAAV9-shRNA was utilized to knockdown ANKRD1 in mouse kidney. **B and C.** Representative immunoblot images of ACSL1 and ACSL4 in H_2O_2 treated HK-2 cells. Lv-FLAG-ANKRD1 and Lv-shANKRD1 were utilized to overexpress or knockdown ANKRD1 in HK-2 cells respectively. n.s. no significance.

Fig. S3. ANKRD1 expression is elevated in renal tubular epithelial cells in renal IRI.
A and **B.** The scRNA-seq data revealed that the expression of ANKRD1 in proximal tubular epithelial cells (PT S3 and S1) of mouse kidneys significantly increased after experiencing short (23 min) or long (30 min) bilateral ischemia. scRNA-seq data are obtained from Susztaklab Kidney Biobank: Mouse Kidney IRI scRNA-seq (https://susztaklab.com/Mouse_IRI_scRNA/index.php). Experimental grouping: Male C57BL/6 mice were subjected to short and long ischemia and followed for 1, 3 and 14 d. Bulk and scRNA-seq was performed in n=2 mice per condition and time point and compared to n = 6 controls. **C** and **D.** Serum SCr and BUN indicated a significant decrease of renal function in renal I/R-treated mice. **E.** Quantitative analysis of renal tubular epithelial cell damage. **F.** Immunoblot analysis of ANKRD1, KIM-1 and NGAL in H_2O_2-treated HK-2 cells with β-actin as a loading control. ***$P < 0.001$.

Fig. S4. Knockdown of ANKRD1 in mice via tail vein attenuated IRI-induced kidney injury and ferroptosis. Alterations in the protein levels of KIM-1, ACSL3 and GPX4 were detected by Western blot after tail vein injection of rAAV9 to intervene with ANKRD1 levels in mice kidney.

Fig. S5. ANKRD1 exacerbates H/R-induced renal tubular epithelial cell injury and ferroptosis.
A. Cell viability of HK-2 cells under different treatment conditions was analyzed by CCK-8. **B.** Representative cellular images of ANKRD1 and KIM-1 IF staining. Scale bar, 50 μm.

Fig. S6. ANKRD1 exacerbates H/R-induced GSH depletion, MDA accumulation, and disturbances of anti-ferroptotic proteins in renal tubular epithelial cells.
A. Representative immunoblot images and quantification of GPX4, FSP1, HO-1, and SOD2 in HK-2 cells. **B** and **C.** Intracellular MDA and GSH levels of HK-2 cells with different treatments. ***$P < 0.001$.

Fig. S7. ANKRD1 interacts with ACSL3 in H/R-treated HK-2 cells. H/R-treated HK-2 cells lysates were immunoprecipitated with IP with IgG, ANKRD1, and ACSL3 antibody, respectively, followed by ANKRD1 and ACSL3 immunoblotting.

Fig. S8. ANKRD1 decreases ACSL3 expression and aggravates ferroptosis in H/R-treated HK-2 cells. A. Representative immunoblot images and quantification of ACSL3, GPX4, FSP1, HO-1, and SOD2 in H/R-treated HK-2 cells after overexpression or knockdown of ACSL3. **B** and **C.** Detection of MDA and 4-HNE in H/R-treated HK-2 cells after intervention with ACSL3. **D** and **E.** Effects of overexpression or knockdown of ANKRD1 on ACSL3 in H/R-treated HK-2 cells as determined by western blot. **F.** Representative immunoblot images of ACSL3, GPX4, FSP1 and HO-1 in H/R-treated HK-2 cells after overexpression of ANKRD1 and ACSL3. **G.** Representative immunoblot images and quantification of ACSL3, GPX4, FSP1 and HO-1 in H/R-treated HK-2 cells after knockdown of ANKRD1 and ACSL3. **$P < 0.01$, ***$P < 0.001$.

Fig. S9. TRIM25 is implicates in ANKRD1-mediated ubiquitination of ACSL3 in H/R-treated HK-2 cells. A. Detection of endogenous ubiquitination levels of ACSL3 in H/R-treated HK-2 cells. **B.** Representative immunoblot images of ACSL3 in H/R-treated HK-2 cells after knockdown of the indicated genes. **C.** Renal IRI led to an increase in the endogenous binding of ANKRD1 and ACSL3 with TRIM25. **D.** High levels of TRIM25 were observed to augment endogenous ubiquitination of ACSL3 in H/R-stimulated HK-2 cells. Myc tagged TRIM25 was transfected into HK-2 cells, and endogenous ubiquitination levels of ACSL3 were detected by immunoprecipitation and western blot analysis.

Fig. S10. ANKRD1, ACSL3 and TRIM25 colocalize in both the nucleus and the cytoplasm of HK-2 cells. Representative images of triple immunofluorescence staining of ANKRD1/ACSL3/TRIM25 in HK-2 cells.

Fig. S11. Interfering with ANKRD1 levels does have minimal impact on the transcription of ACSL3 and TRIM25 *in vivo* and *in vitro*. Relative mRNA levels of ACSL3 and TRIM25 *in vivo* and *in vitro* after intervention with ANKRD1. ns. no significant.

Fig. S12. Knockdown of TRIM25 alleviates ferroptosis caused by ANKRD1 in H/R-treated HK-2 cells.
A. Representative immunoblot images of ACSL3, GPX4, FSP1, HO-1, and SOD2 in

H/R-treated HK-2 cells with different treatments. **B.** MDA levels in H/R-treated HK-2 cells with different treatments. **C.** GSH levels in H/R-treated HK-2 cells with different treatments. $^{**}P < 0.01$, $^{***}P < 0.001$.

Table. S1. Detailed information on molecular docking interaction sites. ACSL3: receptor protein; ANKRD1: ligand protein.

Fig.S1. Altering ANKRD1 expression can not significantly impact P53/SLC7A11 levels in both *in vitro* and *in vivo* IRI models.

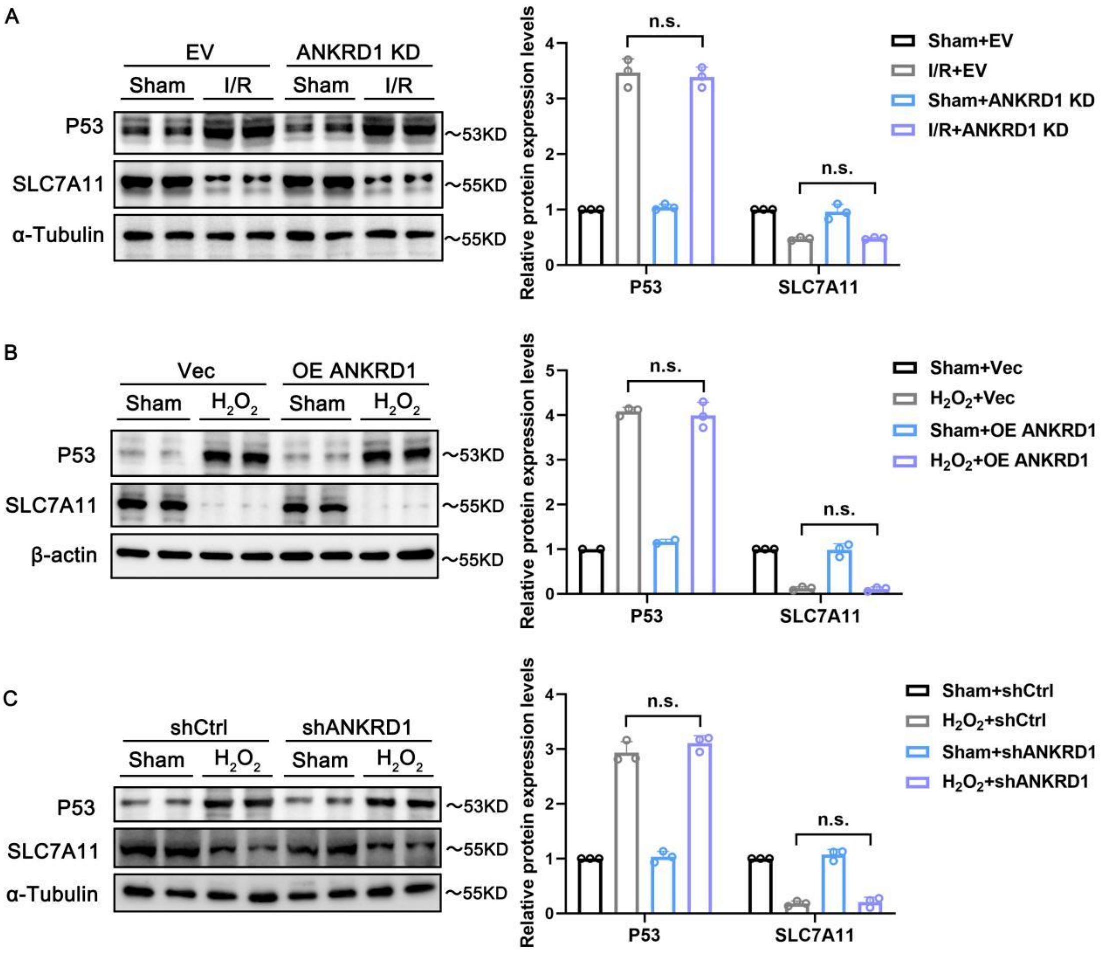

Fig.S2. Altering ANKRD1 expression can not significantly impact ACSL1/4 levels in both *in vitro* and *in vivo* IRI models.

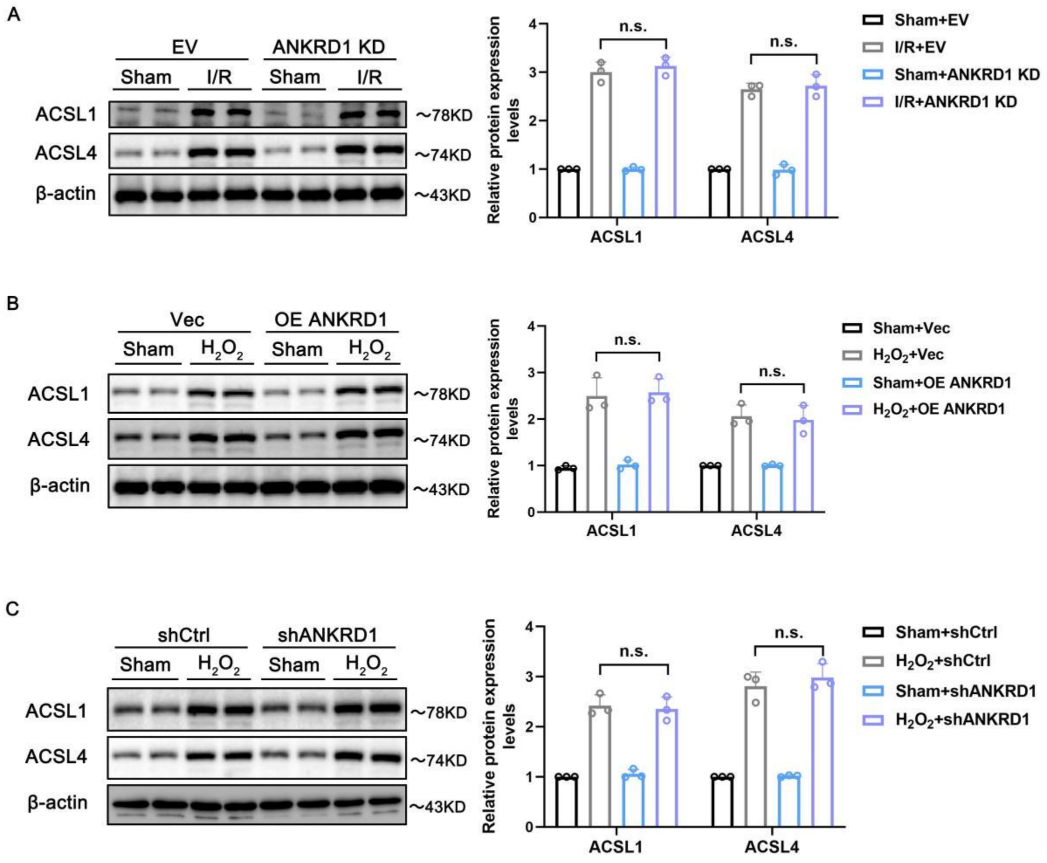

Fig.S3. ANKRD1 expression is elevated in renal tubular epithelial cells in renal IRI.

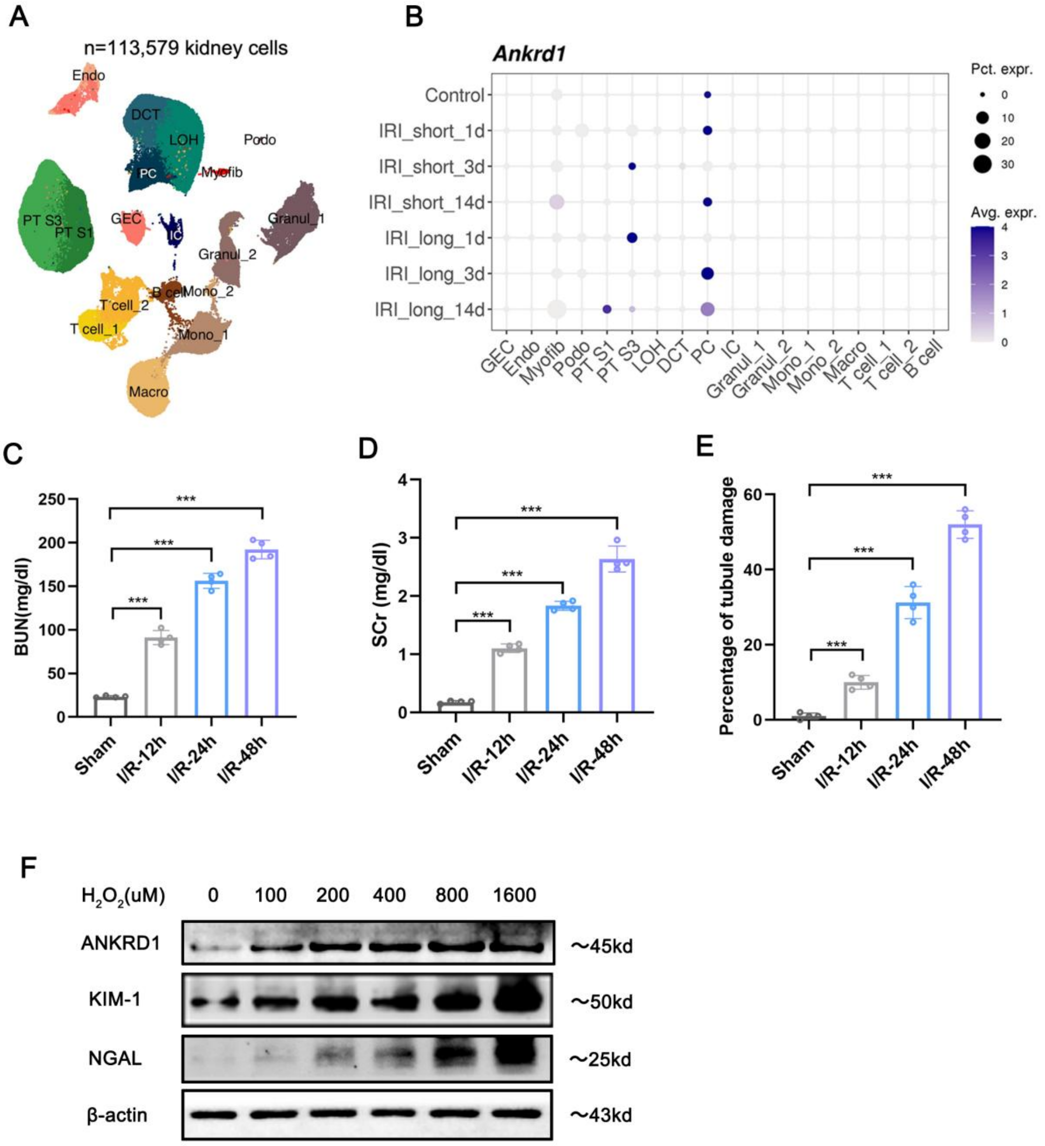

Fig.S4. Knockdown of ANKRD1 in mice via tail vein attenuates IRI-induced kidney injury and ferroptosis.

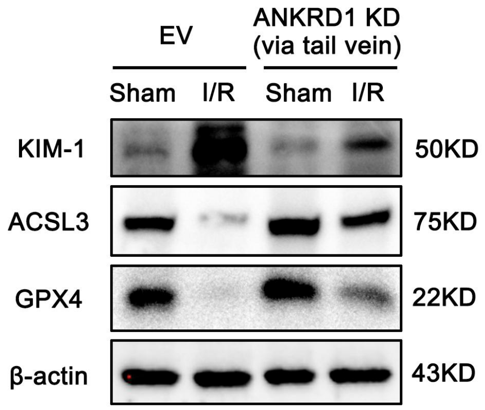

Fig.S5. ANKRD1 exacerbates H/R-induced renal tubular epithelial cell injury and ferroptosis.

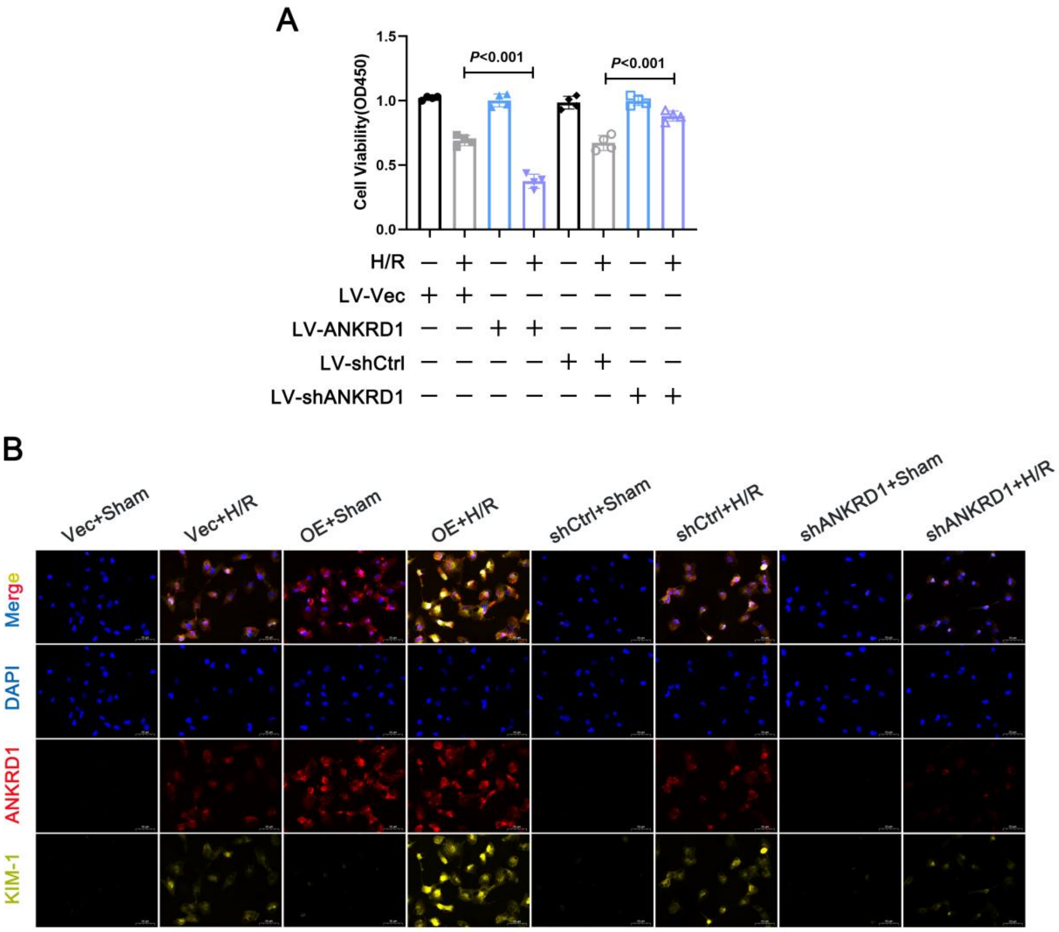

Fig.S6. ANKRD1 exacerbates H/R-induced GSH depletion, MDA accumulation, and disturbances of anti-ferroptotic proteins in renal tubular epithelial cells.

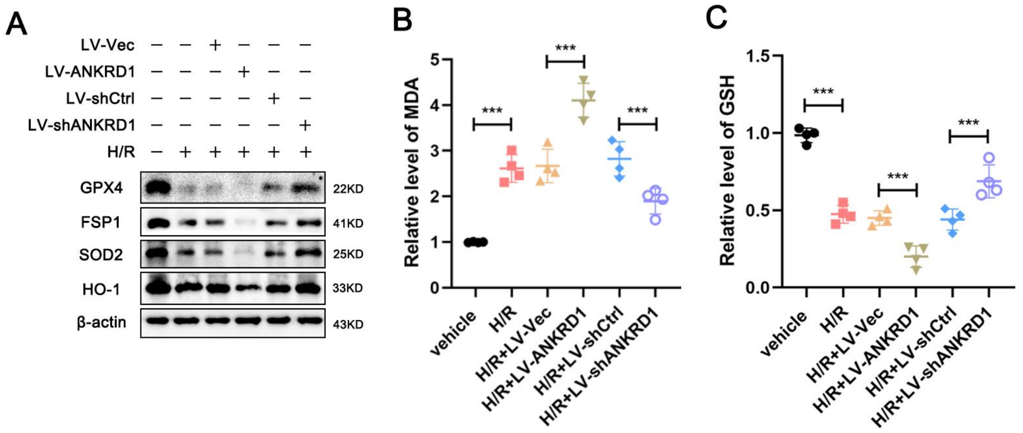

Fig.S7. ANKRD1 interacts with ACSL3 in H/R-treated HK-2 cells.

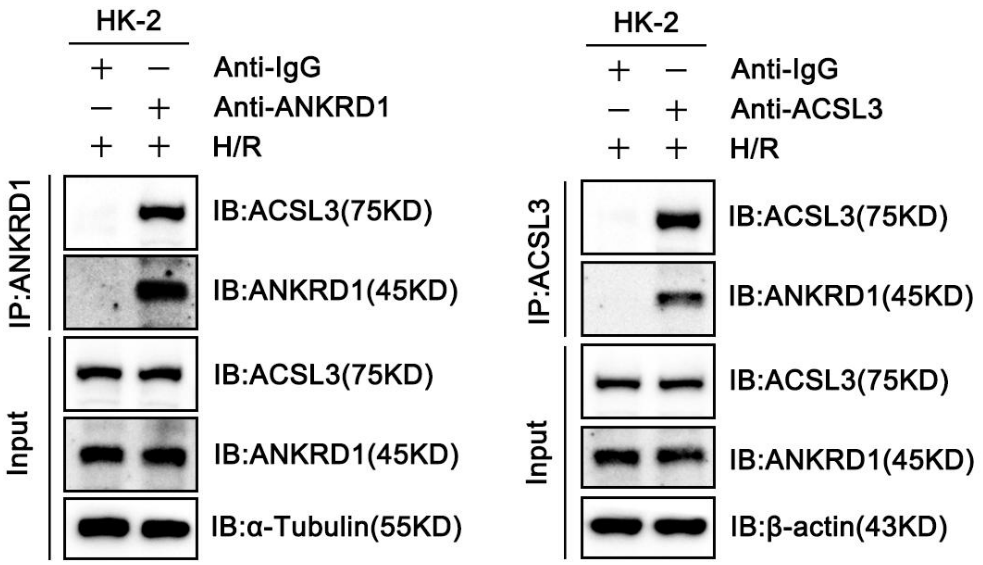

Fig.S8. ANKRD1 decreases ACSL3 expression and aggravates ferroptosis in H/R-treated HK-2 cells.

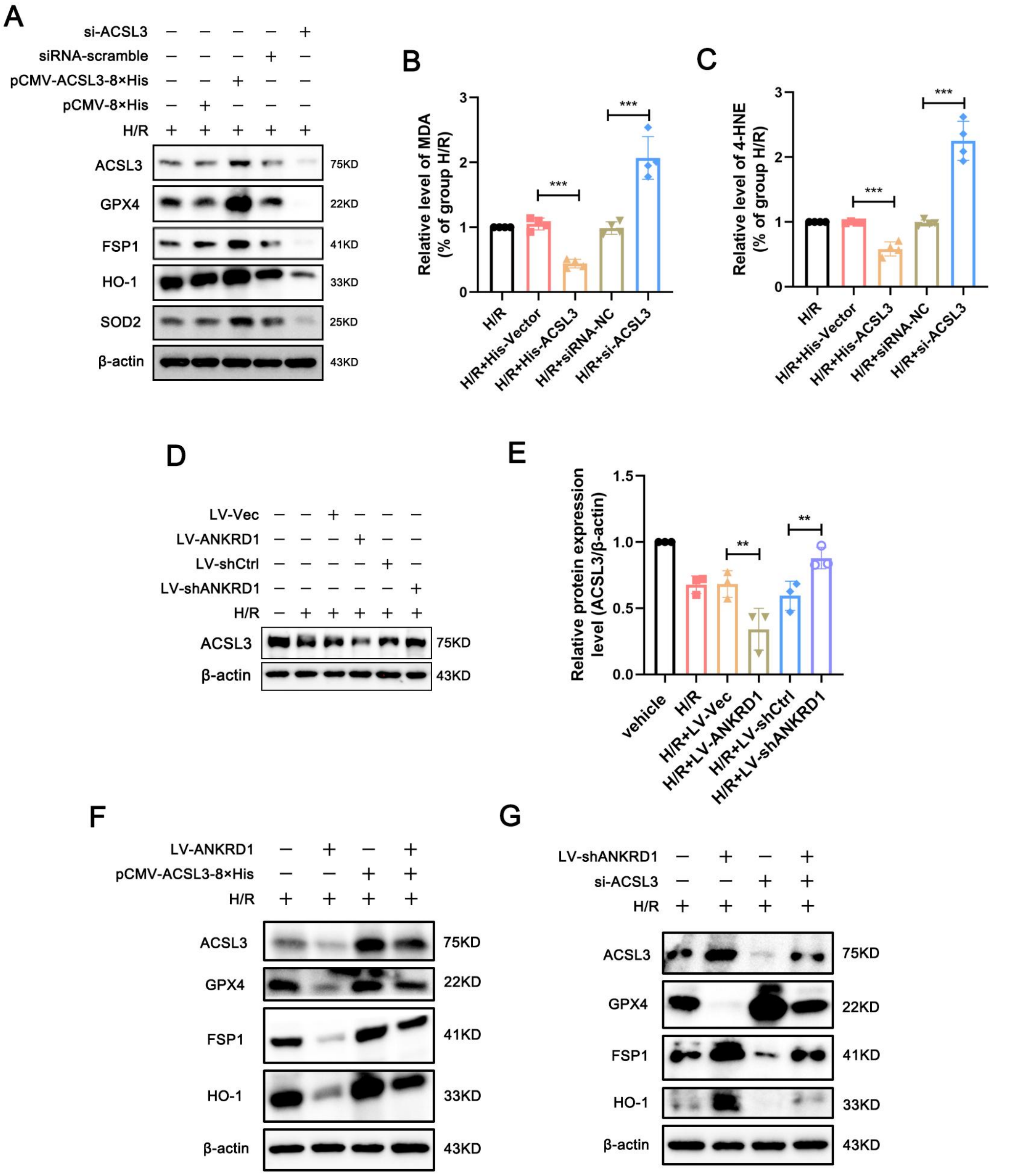

Fig.S9. TRIM25 is implicated in ANKRD1-mediated ubiquitination of ACSL3 in H/R-treated HK-2 cells.

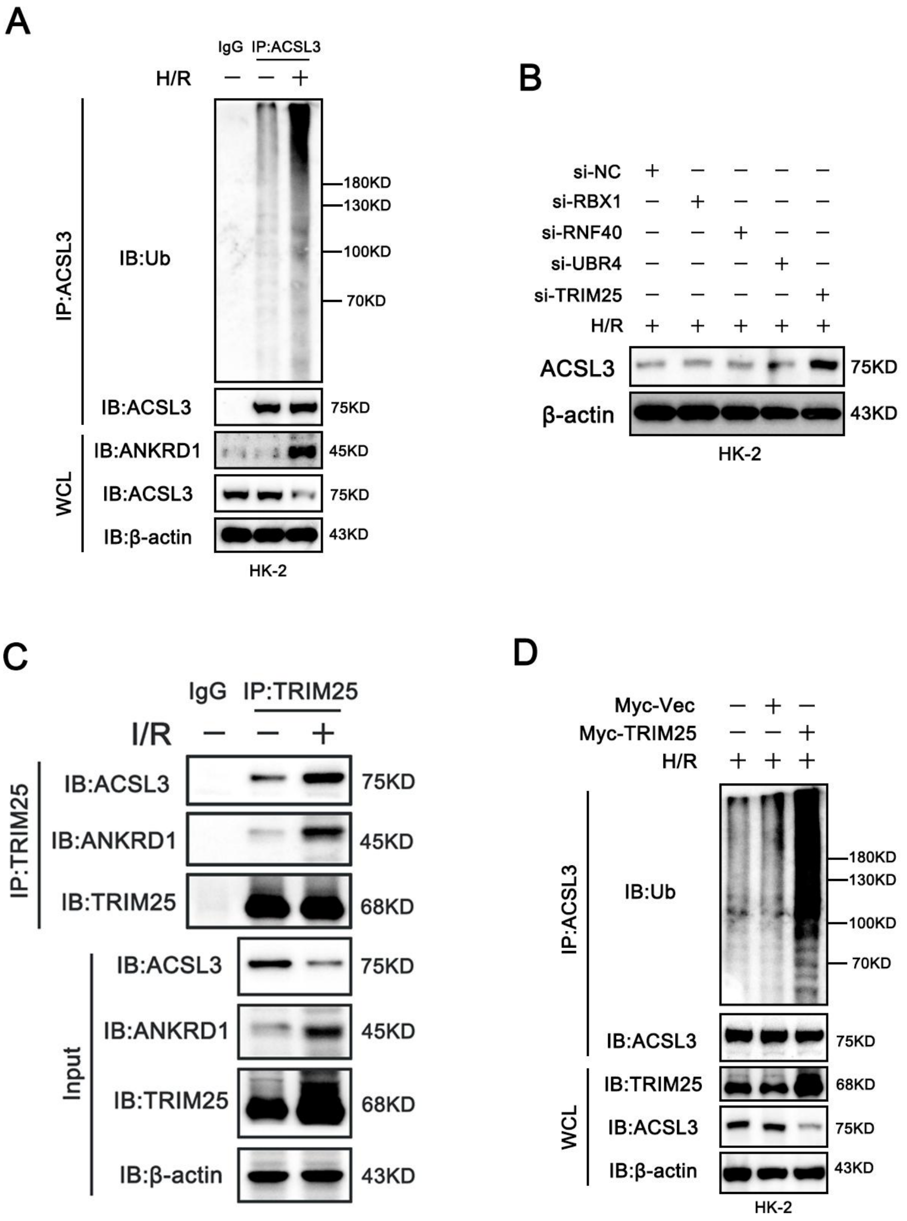

Fig.S10. ANKRD1, ACSL3 and TRIM25 colocalize in both the nucleus and the cytoplasm of HK-2 cells.

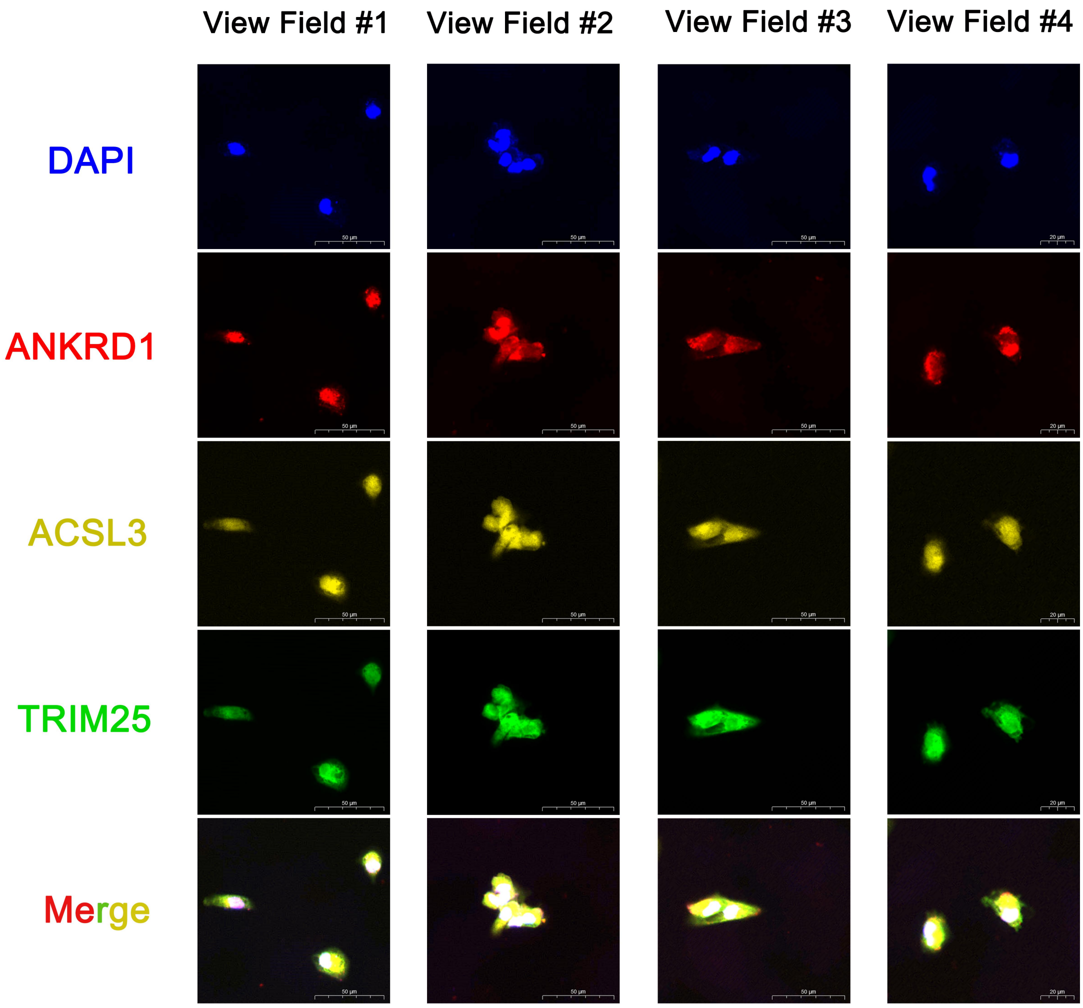

Fig. S11. Interfering with ANKRD1 levels does have minimal impact on the transcription of ACSL3 and TRIM25 *in vivo* and *in vitro*.

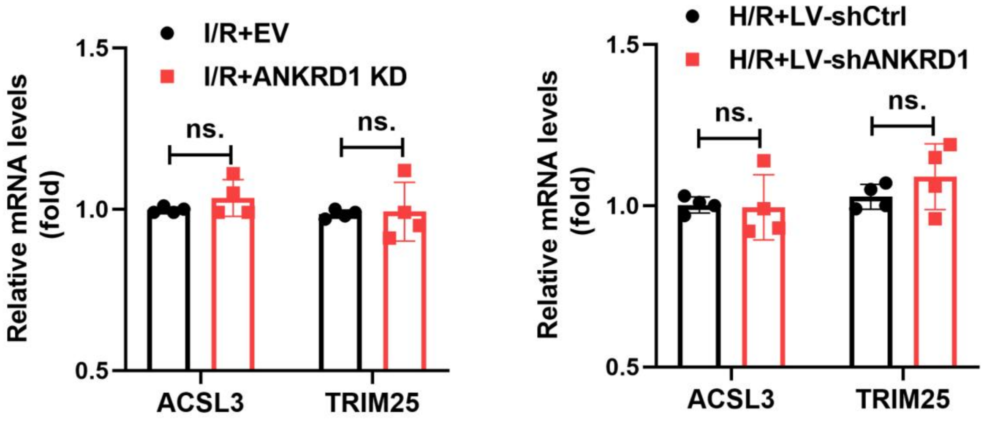

Fig.S12. Knockdown of TRIM25 alleviates ferroptosis caused by ANKRD1 in H/R-treated HK-2 cells.

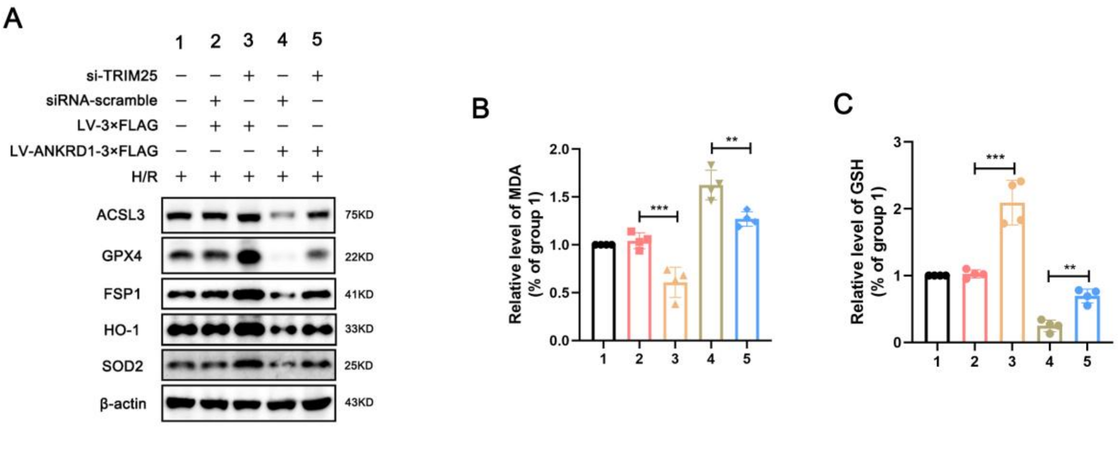

Table. S1 Detailed information on molecular docking interaction sites.

Receptor interface residue(s):		Hydrogen Bonds(Å)
LYS	13A	4.666
PRO	20A	3.569
LEU	23A	2.991
TYR	24A	2.529
HIS	27A	2.856
PHE	28A	3.17
SER	31A	3.317
LEU	32A	3.656
ILE	35A	1.744
TYR	38A	2.871
ILE	39A	3.08
TYR	42A	2.388
PHE	43A	2.983
PRO	83A	3.367
LYS	98A	2.723
PHE	99A	4.185
GLU	418A	4.725
ARG	424A	3.747
LEU	428A	4.867
PHE	432A	2.97
ARG	435A	2.731

Ligand interface residue(s):		Hydrogen Bonds(Å)
LEU	48A	3.656
GLU	57A	2.97
TRP	60A	2.731
ARG	96A	2.723
ARG	100A	3.311
LYS	103A	3.367
TRP	295A	3.947
THR	299A	2.983
ILE	302A	3.909
PHE	303A	2.388
LEU	306A	3.117
ARG	307A	1.744
SER	310A	3.317
TYR	311A	3.17
THR	313A	4.725
SER	314A	2.856
ARG	315A	2.864
ALA	317A	3.874
THR	318A	2.529
PHE	319A	2.869

Receptor-ligand interface residue pair(s):			Hydrogen Bonds(Å)
13A	-	317A	4.666
20A	-	318A	3.569
23A	-	317A	3.999
23A	-	318A	2.991
24A	-	314A	4.168
24A	-	315A	2.864
24A	-	318A	2.529
24A	-	319A	2.869
27A	-	314A	2.856
27A	-	315A	4.846
27A	-	317A	3.874
27A	-	318A	4.05
28A	-	311A	3.17
28A	-	314A	4.707
31A	-	310A	3.317
31A	-	311A	3.484
31A	-	314A	3.763
32A	-	311A	3.656
35A	-	307A	1.744
35A	-	310A	4.25
35A	-	311A	3.696
38A	-	303A	2.871
38A	-	306A	3.117
39A	-	303A	3.08
39A	-	307A	3.179
42A	-	299A	3.329
42A	-	302A	3.909
42A	-	303A	2.388
43A	-	295A	3.947
43A	-	299A	2.983
43A	-	303A	3.678
83A	-	103A	3.367
98A	-	96A	2.723
98A	-	100A	3.311
99A	-	96A	4.185
418A	-	313A	4.725
424A	-	306A	3.747
428A	-	48A	4.867
432A	-	48A	3.656
432A	-	57A	2.97
435A	-	60A	2.731
