# Supplementary material for: ANKRD1 aggravates renal ischaemia‒reperfusion injury via promoting TRIM25‐mediated ubiquitination of ACSL3
Source: Clin Transl Med. 2024 Sep 17;14(9):e70024. doi: 10.1002/ctm2.70024 (PMC11406046; doi:10.1002/ctm2.70024)
Supplement: Supplementary file 2 — Supporting information [file CTM2-14-e70024-s001.docx]

**Supplementary Materials and Methods**

**Construction of renal IRI model**

The bilateral renal pedicles were clamped using microaneurysm clips for 30 minutes. Sham-operated mice underwent the same surgical procedure without clamping. Mice were sacrificed after reperfusion at various times (6/12/24/48h), and kidney and serum samples were collected as required for the experiments. To inhibit ACSL3 levels *in vivo*, Triacsin C (10 mg/kg/d; p.o.; 3 weeks) was administered to mice every two days. All procedures involving the mice were carried out according to the National Institutes of Health Guide for the Care and Use of Laboratory Animals and authorized by the Ethical Committee for Animal Experimentation, Renmin Hospital of Wuhan University (approval no. WDRM20230304B).

**Renal function measurement**

Whole blood specimens were collected and centrifuged at 500g for 15 min at 2–8 °C. Serum was taken and tested. Serum creatinine and blood urea nitrogen (BUN) levels were measured using a creatinine assay kit (BioAssay Systems, Hayward, CA, USA) and a Hitachi 7060 automated biochemistry analyzer (Hitachi, Tokyo, Japan) in accordance with the manufacturer’s instructions.

**Renal morphological analysis**

Fresh mouse kidney tissues were fixed in 4% paraformaldehyde for over 24 h. The

fixed kidney tissues were dehydrated, embedded in paraffin wax, and sectioned to

have 4 μm thickness via conventional methods. Then, the paraffin sections were dewaxed, hydrated, and stained with hematoxylin and eosin (H&E). Histological results were examined using the Paller semi-quantitative scoring system in a blinded

manner.

**TUNEL staining**

Tissues were embedded in paraffin, cut into slices, dewaxed, dehydrated, and incubated with proteinase K for 15 min at 37 ℃ . The sections were incubated for 10 min using a film-breaking working solution and washed with PBS. The tissues were incubated with a TUNEL reaction mixture for 60 min at 37 °C. Next, the nuclei were stained with DAPI, and the slices were sealed with an antifluorescein quencher. Each tissue section was observed under a fluorescent microscope, and 10 fields of view

were randomly selected to count TUNEL-positive cells.

**Immunofluorescence**

Paraffin renal sections were dewaxed and hydrated, then stained for ANKRD1 and KIM- 1. First, EDTA high-temperature repair was performed for 20 min, then mouse

kidney sections were blocked with 10% BSA, followed by incubation at 4 ℃ overnight with respective corresponding primary antibodies: anti-ANKRD1 (1:2000; cat. no. 67775- 1-Ig, Proteintech group), anti-KIM- 1 (1:500; cat. no. PA5-79345, Thermo Fisher Scientific). The sections were then washed with PBS and incubated with secondary antibody (Alexa Flour® 568 goat anti-Rabbit IgG(H+L) A11036, Invitrogen, Thermo Fisher Scientific) at 37 ℃ for 1 h. Nuclei were stained with DAPI.

For cell immunofluorescence, cover glass was added to the six‐well plate in advance, and the cells were plated until they grew to an appropriate density. The medium was then removed and the cells were fixed in 4% paraformaldehyde for 30 min. Finally, the subsequent operation is the same as that in tissue immunofluorescence. Primary antibodies: anti-ANKRD1 (1:2000; cat. no. 67775- 1-Ig, Proteintech group), anti-KIM- 1 (1:500; cat. no. PA5-79345, Thermo Fisher Scientific), anti-TRIM25 (1:500; cat. no. 12573- 1-AP, Proteintech group) and anti-ACSL3 (1:500; cat. no. A22085, ABclonal).

**Cell viability assay**

Cell viability was determined using Cell Counting Kit-8 (Biosharp, China) in accordance with the manufacturer’s instructions. HK-2 cells were placed in 96-well plates at a density of 5 × 103 cells/well and treated with various conditions. Each well was incubated with 10 μl of CCK-8 solution at 37 ℃ for 1 h, and the absorbance of each well was measured at 450 nm using a microplate reader. Each group was replicated and measured three times.

**Western blot analysis**

Tissues and cells were lysed with RIPA lysate, PMSF, and phosphatase inhibitor cocktail (Beyotime Biotechnology, China) in an ice bath for 30 min. The supernatant was collected by centrifugation at 8000g for 10 min at 4 °C, and the protein concentration of each sample for each group was measured three times by using a BCA protein assay kit (Beyotime Biotechnology, China). Then, 30 µg of proteins in

each group were separated via SDS-PAGE and transferred to PVDF (EMD Millipore,

USA) membranes. The PVDF membranes were then blocked with 5% skim milk for 1 h at room temperature and incubated overnight at 4 °C in respective corresponding primary antibodies: anti-ANKRD1 #1 (1:2000; cat. no. 67775- 1-Ig, Proteintech group), anti-ANKRD1 #2 (1:1000; cat. no. sc-365056, Santa Cruz), anti-KIM- 1 (1:500; cat. no. PA5-79345, Thermo Fisher Scientific), anti-NGAL (1:1000; cat. no. 26991- 1-AP, Proteintech group), anti-TRIM25 (1:500; cat. no. 12573- 1-AP, Proteintech group), anti-ACSL3 (1:1000; cat. no. 30214- 1-AP, Proteintech group), anti-GPX4 (1:1000; cat. no. 30388- 1-AP, Proteintech group), anti-FSP1 (1:2000; cat. no. 20886- 1-AP, Proteintech group), anti-HO- 1 (1:2000, cat. no. 10701- 1-AP, Proteintech group), anti-SOD2 (1:5000, cat. no. WL02506, Wanleibio), anti-Myc (1:2000; cat. no. 16286- 1-AP, Proteintech group), anti-His (1:5000; cat. no. 66005- 1-Ig, Proteintech group), anti-FLAG #1 (1:5000; cat. no. 66008-4-Ig, Proteintech group), anti-FLAG #2 (1:1000; cat. no. F1804, Sigma-Aldrich), anti-HA (1:5000; cat. no. 51064-2-AP, Proteintech group), anti-Ub (1:1000; cat. no. 10201-2-AP, Proteintech group), anti- β-actin (1:1000; cat. no. 66009- 1-Ig, Proteintech group), anti-α-Tubulin (1:20000; cat. no. 66031- 1-Ig, Proteintech group), anti-GAPDH (1:50000; cat. no. 60004- 1-Ig, Proteintech group). The next day, the membranes were incubated in secondary antibody (Cell Signaling Technology, USA) for 1 h and visualized using an ECL kit (Bioshap, China) on a ChemiDoc MP imaging system (Bio-Rad, USA). The experiment was repeated three times for each group. All target proteins were quantified using Image J software in comparison with the internal reference.

**Measurement of Intracellular GSH**

Intracellular GSH levels were measured using the GSH Assay Kit (Nanjing Jiancheng Bioengineering Institute, China). Briefly, cells from each treatment group were collected and centrifuged to obtain the supernatant. The working solution was added to the supernatant and then centrifuged at 800 × g for 10 min. The mixture (1 ml) was collected for color development reaction in accordance with the instructions. Each treatment group (100 μl) was collected and placed in a 96-well plate, and the absorbance was measured at 420 nm.

**Lipid ROS Assessment**

Relative lipid reactive oxygen levels in cells were assessed using the C11 BODIPY 581/591 lipid peroxidation fluorescent probe (Maokangbio, MX5211- 1MG, China). The cells of each group were incubated with 5 μM C11-BODIPY for 30 min at 37 °C. Images were acquired and analyzed using an orthofluorescence microscope. Reduced state dyes were measured with Ex/Em = 581/591 nm (Texas Red filter), and oxidized dyes were measured with Ex/Em = 488/510 nm (FITC filter).

**Total Reactive oxygen species detection**

Intracellular ROS assay was performed using 2′, 7′-dichlorodihydrofluorescein diacetate (DCFHDA) staining (Beyotime, China) as previously described. Briefly, cells were stained with 10 μM DCFH-DA in serum-free DMEM medium for 30 min and imaged under a fluorescence microscope. Besides, ROS were detected and quantified through flow cytometry (BD Biosciences, USA).

**Malondialdehyde (MDA) and 4-Hydroxynonenal (4-HNE) Assay**

The contents of MDA and 4-HNE was measured to reflect the accumulation of lipid peroxidation in tissues and cells, which indirectly indicated the degree of cellular damage. MDA and 4-HNE levels in each group of renal tissues or cells were measured using the MDA and 4-HNE kit (Nanjing Jiancheng Bioengineering Institute, China) in accordance with the manufacturer's protocol. Each group was replicated and

measured thrice.

**Cell transfection**

Stably transfected cell lines were constructed by transferring ANKRD1 overexpression and knockdown lentivirus (Lv) into HK-2 and HEK 293T, respectively, according to the manufacturer's instructions (Genechem). The component order of the overexpression vector was Ubi-MCS-3FLAG-CBh-gcGFP-IRES-puromycin; the knockdown vector component sequence was hU6-MCS-CBh-gcGFP-IRES-puromycin, and the target sequence in human: GAATGGAACCAAAGCAATA.

Human expression plasmids encoding FLAG-tagged ANKRD1, His-tagged ACSL3, Myc-tagged TRIM25, HA-tagged ubiquitin, HA-tagged ubiquitin-K48, and HA-tagged ubiquitin-K63 were purchased from Miaoling Biology. For transient transfection, plasmids were transfected into HK-2 or HEK 293T cells with Lipofectamine™ 2000 transfection reagent as required for the experiment.

The siRNAs for *in vitro* transfection were obtained from OBiO Technology (Shanghai, China). si-ACSL3: GCTGTGTAACAGTTGTGAAAT; si-RBX1: TGGGATATTGTGGTTGATAAC; si-RNF40: CGCATCGAGTTTGAGCAGAAT; si-UBR4: CCACATACATTGTTCGGGAAA; si-TRIM25 #1: GAACTGAACCACAAGCTGATA; si-TRIM25 #2: GAGTGAGATCCAGACCTTGAA; si-TRIM25 #3: CCGGAACAGTTAGTGGATTTA. HK-2 and HEK 293T cells were transfected with siRNAs via Lipofectamine™ 2000 transfection reagent according to the manufacturer’s recommendations.

### **Library construction and RNA-seq**

Mouse kidneys receiving I/R treatment and controls (n=3) were collected for sequencing. Total RNAs were extracted from mouse kidney tissue using TRIzol Reagent (Invitrogen, cat. NO 15596026) according to the protocols described by Chomczynski et al. [26]. DNA digestion was performed after RNA extraction by DNaseI. RNA quality was verified by measuring A260/A280 using a NanodropTM OneCspectrophotometer (Thermo Fisher Scientific Inc). 1.5% agarose gel electrophoresis was performed to validate RNA integrity. At last, qualified RNAs were quantified by Qubit3.0 with QubitTM RNA Broad Range Assay kit (Life Technologies, Q10210).

2 μg total RNAs were used to prepare stranded RNA sequencing library using KCTM Stranded mRNA Library Prep Kit for Illumina (Catalog NO. DR08402, Wuhan Seqhealth Co., Ltd. China) according to the manufacturer’s instruction. PCR products corresponding to 200-500 bps were enriched, quantified and finally sequenced on Novaseq 6000 sequencer (Illumina) with PE150 model.

**Co-immunoprecipitation (Co-IP) and ubiquitination assays**

The desired plasmids or lentivirus were first co-transfected into HK-2 or HEK 293T cells, and the cells were lysed with IP lysis buffer (50 mM Tris-HCl, pH 7.4; 150 mM NaCl; 1 mM EDTA-2Na; 1% Triton X-100; 1% sodium deoxycholate and 0.1% SDS) after required specific transfection time. The samples were incubated with 2~5 μg antibodies (IgG/ACSL3/FLAG/His/Myc) overnight at 4°C. Protein A/G beads were then added and incubated at 4°C overnight. The beads were washed three times with elution buffer and resuspended with SDS loading buffer before being heated at 95℃ for 10 min, and then Western blot analysis was performed.

For ubiquitination analysis, the desired plasmids or lentivirus were first co-transfected into HK-2 or HEK 293T cells. Lysates were precipitated with anti-His or ACSL3 (endogenous ubiquitination) antibody, and the IP procedure was repeated as described above. Immunoblotting was performed with anti-HA or Ub antibody to measure ubiquitination levels.

**IP-Mass spectrometry (IP-MS)**

HEK 293 cells (1 × 10^8^) were transfected with Lv-FLAG-ANKRD1 (human). FLAG-tagged ANKRD1 was immunoprecipitated and desalted. MS analysis was performed by SpecAlly (Wuhan) Life Science and Technology Company.

**Sample preparation**

Beads samples were incubated in the reaction buffer (1% SDC/100 mM Tris-HCl, pH 8.5/10 mM TCEP/40 mM CAA) at 95 °C for 10 min for protein denaturation, cysteine reduction and alkylation. The eluates were diluted with equal volume of H_2_O and subjected to trypsin digestion overnight by adding trypsin at a ratio of 1:50 (enzyme: protein, w/w) for overnight digestion at 37 °C. The next day, TFA was used to bring the pH down to 6.0 to end the digestion. After centrifugation (12000×g, 15 min), the peptide was purified using self-made SDB desalting columns. The eluate was vacuum dried and stored at -20 °C for later use.

**LC-MS/MS Detection**

LC-MS/MS data acquisition was carried out on a Q Exactive HF mass spectrometer coupled with UltiMate 3000 RSLCnano system. Peptides were loaded through auto-sampler and separated in a C18 analytical column (75μm × 25cm, C18, 1.9μm, 100Å). Mobile phase A (0. 1% formic acid) and mobile phase B (80% ACN, 0. 1% formic acid) were used to establish the separation gradient. A constant flow rate was set at 300 nL/min. For DDA mode analysis, each scan cycle consisted of one full-scan mass spectrum (R = 60 K, AGC = 3e6, max IT = 20 ms, scan range = 350–1800 m/z) followed by 20 MS/MS events (R = 15 K, AGC = 2e5, max IT = 50 ms). HCD collision energy was set to 28. The isolation window for precursor selection was set to 1.6 Da. Former target ion exclusion was set for 30 s.

**Database Search and Data Analysis**

MS raw data were analyzed with MaxQuant (V1.6.6) using the Andromeda database search algorithm. Spectra files were searched against Human database(2022-03-29, 20377 entries) using the following parameters: LFQ mode was checked for quantification; Variable modifications, Oxidation (M), Acetyl (Protein N-term) & Deamidation (NQ); Fixed modifications, Carbamidomethyl (C); Digestion, Trypsin/P; The MS1 match tolerance was set as 20 ppm for the first search and 4.5 ppm for the main search; the MS2 tolerance was set as 20 ppm. Search results were filtered with 1% FDR at both protein and peptide levels. Proteins denoted as decoy hits, contaminants, or only identified by sites were removed, and the remaining identifications were used for further quantification analysis. Proteins with a fold change > 4 between bait IP and control were screened out as interactors of the bait protein.

**Molecular Docking**

The protein models used for docking were ANKRD1 (AlphaFold Protein Structure Database: Q15327) and ACSL3 (AlphaFold Protein Structure Database: O95573). HDOCK SERVER (http://hdock.phys.hust.edu.cn/) was used for molecular docking. ACSL3 was selected as the receptor protein and ANKRD1 was selected as the ligand protein. Pre-treatment of the proteins (deletion of water molecules and excess ligands, addition of hydrogen atoms) was completed using PyMol 2.4. The model with the lowest binding energy was selected as the best docking model, and PyMOL was used to visualise protein-protein interactions.

**Proximity ligation assay (PLA)**

The Duolink® PLA Multicolor Probemaker Kit (Sigma-Aldrich, Shanghai, China) was utilized to measure protein-protein interactions in HK-2 cells in accordance with the manufacturer's protocol. In brief, the fixed samples were blocked in an incubator. The corresponding primary antibody was added for incubation. Subsequently, PLA working solution was prepared for ligation and amplification. After adding the detection solution, the samples were washed and sealed, and images were captured under a fluorescence microscope.

**Reagents**

MG-132 (CAS no. 133407-82-6), Cycloheximide (CHX, CAS no. 66-81-9), and Triacsin C (CAS no. 76896-80-5) were purchased from MedChemExpress (Shanghai, China). MG-132 and CHX were all dissolved in DMSO (final concentration of less than 0. 1% (v/v)). Coomassie Blue Fast Staining and No-decoloring Solution (CAS no. PS111) and Protein A/G Magnetic Beads (CAS no. YJ003) were purchased from Epizyme Biotech (Shanghai, China).

**Statistical analysis**

Statistical analysis was conducted using GraphPad Prism 8.3.0, and the data are presented as the mean ± SEM. Student t-test was used to establish statistical significance between two groups, and one-way ANOVA followed by Dunnett’s post-hoc test was applied among groups of more than two. Correlation analysis was performed using Pearson correlation statistical analysis. *P*<0.05 was considered statistically significant.

**Data availability**

RNA-seq datasets are available from Gene Expression Omnibus database (https://www.ncbi.nlm.nih.gov/geo, under accession numbers GSE39548, GSE71647, GSE192532, GSE98622). scRNA-seq data are obtained from Susztaklab Kidney Biobank: Mouse Kidney IRI scRNA-seq (https://susztaklab.com/Mouse_IRI_scRNA/index.php). All remaining data are presented within the article and information files, and available from corresponding author upon request.
